# PTRE-seq reveals mechanism and interactions of RNA binding proteins and miRNAs

**DOI:** 10.1038/s41467-017-02745-0

**Published:** 2018-01-19

**Authors:** Kyle A. Cottrell, Hemangi G. Chaudhari, Barak A. Cohen, Sergej Djuranovic

**Affiliations:** 1 Department of Cell Biology and Physiology, School of Medicine, Washington University, St. Louis, MO 63110 USA; 2 Department of Genetics, School of Medicine, Washington University, St. Louis, MO 63110 USA; 3 The Edison Family Center for Genome Sciences and Systems Biology, School of Medicine, Washington University, St. Louis, MO 63110 USA

## Abstract

RNA binding proteins (RBP) and microRNAs (miRNAs) often bind sequences in 3′ untranslated regions (UTRs) of mRNAs, and regulate stability and translation efficiency. With the identification of numerous RBPs and miRNAs, there is an urgent need for new technologies to dissect the function of the *cis*-acting elements of RBPs and miRNAs. We describe post-transcriptional regulatory element sequencing (PTRE-seq), a massively parallel method for assaying the target sequences of miRNAs and RBPs. We use PTRE-seq to dissect sequence preferences and interactions between miRNAs and RBPs. The binding sites for these effector molecules influenced different aspects of the RNA lifecycle: RNA stability, translation efficiency, and translation initiation. In some cases, post-transcriptional control is modular, with different factors acting independently of each other, while in other cases factors show specific epistatic interactions. The throughput, flexibility, and reproducibility of PTRE-seq make it a valuable tool to study post-transcriptional regulation by 3′UTR elements.

## Abstract (web summary)

A large number of RNA binding proteins (RBPs) and miRNAs bind to the 3′ untranslated regions of mRNA, but methods to dissect their function and interactions are lacking. Here the authors introduce post-transcriptional regulatory element sequencing (PTRE-seq) to dissect sequence preferences, interactions and consequences of RBP and miRNA binding.

## Introduction

Cellular factors post-transcriptionally regulate mRNA by altering its modification, localization, stability, and translation^1^. These *trans*-acting factors often bind to *cis* elements within the mRNA. Two important classes of *trans*-acting factors are RNA binding proteins (RBPs) and microRNAs (miRNAs).

miRNAs are short non-coding RNAs that mediate translational repression and destabilization of target mRNAs^2–9^. miRNAs recruit the Argonaute containing miRNA-induced silencing complex (miRISC) to specific mRNAs by base-pairing with complementary sequences within their 3′UTR^2^. Mammalian cells typically express many miRNAs, with the human genome currently thought to encode 2580 miRNAs^10^. Those miRNAs are predicted to target most human mRNAs^11^.

RBPs are a second prominent class of *trans*-acting factors that affect mRNAs through processes including: splicing, adenylation and deadenylation, degradation, localization, and translation^1^. Recent studies have sought to identify the complete set of RBPs in mammalian cells, and based on these studies the human genome contains >1000 RBPs, most of which have unknown functions^12–15^. Over 800 RBPs have been identified in cultured HeLa cells alone^12^. One well-characterized RBP is Pumilio, a member of the Puf family, which is conserved from yeast to humans and regulates translation and RNA-decay^16–19^. The RNA binding protein Smaug is also conserved from yeast (Vts1) to humans (SAMD4A and SAMD4B) and regulates both translation and RNA-decay^20,21^. Another well-known RBP family is the ELAVLs, homologs of the *Drosophila* embryonic lethal abnormal vision, *elav*^22^. These proteins bind AU-rich elements within mRNAs and either stabilize or destabilize mRNAs, as well as enhance or repress translation^22^. While the function and mechanism of action of some RBPs have been partially elucidated, for the majority of RBPs their functions remain unknown.

Most evidence for the function of RBPs, such as Pumilio and Smaug, has come from low-throughput experiments that study their targets during embryogenesis or from reporter experiments^16,17,19–21^. Given the large numbers of uncharacterized RBPs and miRNAs, we urgently need new approaches with higher throughput, which can be employed in diverse cell types and developmental stages.

Interactions between the miRISC and RBPs have been of great interest recently. With >2500 human miRNAs, that are predicted to target most mRNAs, and >1000 RBPs it is likely that many mRNAs are co-regulated by these factors^10,12–15^. Many RBP or miRNA binding sites have been shown to occur near predicted miRNA binding sites. In many cases these binding sites are immediately adjacent or even overlap^23–28^. Some RBPs cooperate with miRNAs in regulating the expression of specific genes. For example, Pumilio facilitates miRNA-mediated repression in both humans and *Drosophila*^2,29,30^. HuR, a RBP that binds AU-rich elements, can also modulate miRNA-mediated repression^2,31,32^. Understanding how mRNA *trans*-acting factors modulate the activity of one another is a major challenge. A tractable high-throughput approach would help unravel the interactions between different effectors of RNA regulation.

The widespread availability of high-throughput sequencing is powering the development of “omic” technologies to study miRNAs and RBPs. RNA-seq combined with ribosome profiling can reveal the effects of RBPs and miRNAs on target RNA expression and translation^33–35^. While these methods provide the throughput required to study the effects of miRNAs and RBPs across the genome, they do not provide the flexibility to construct and assay large numbers of reporters designed to dissect the effects of different combinations and affinities of RNA *cis*-regulatory elements.

In studies of transcriptional enhancers, Massively Parallel Reporter Gene Assays (MPRAs) are useful complements to technologies that quantify the activity of endogenous genomic elements^36–39^. An analogous technology for assaying the activities of the *cis*-acting RNA sequences bound by RBPs and miRNAs would help unravel the network of interactions that underlies post-transcriptional regulation of mRNA. Such a system should provide the flexibility and throughput to dissect individual 3′ UTR elements, assay the effects of changes in the strength and number of *cis*-acting RNA elements, and detect interactions between different types of *cis*-acting sequences.

Recently, several labs have employed plasmid or mRNA libraries to study endogenous 3′UTR elements^40–43^. These approaches generally rely on synthesizing or amplifying portions of 3′UTRs and fusing them to a reporter. While these techniques have identified 3′UTR motifs that have effects on RNA stability and protein amounts, none have been combined with polysome profiling to separate effects on RNA stability, translation efficiency, and translational initiation. In addition, naturally occurring 3′ UTRs contain many different types of elements, making it difficult to deconvolve the effects of individual sites. A synthetic approach, in which large numbers of reporters with specific combinations of elements are designed and assayed, would provide the power necessary to isolate the effects of individual binding sites, as well as the interactions between sites. Because high-throughput methods for studying synthetic elements have proven to have great utility in dissecting interactions among transcription factors, we have extended this approach to post-transcriptional regulation^44–47^.

Here we report post-transcriptional regulatory **e**lement sequencing (PTRE-seq), an approach that uses a massively parallel reporter library to study the effects of synthetic 3′UTR elements on RNA stability, translation efficiency, and translation initiation. We use PTRE-seq to study the effects of known binding sites for RBPs and miRNAs, both individually and in combination. With this approach, we determine that the binding sites for these effector molecules influenced different aspects of the RNA lifecycle, including RNA stability, translation efficiency, and translation initiation. We observe *trans*-acting factors acting independently or in some cases epistatically. Finally, deploying PTRE-seq across multiple cell lines revealed the influence of the *trans* environment on post-transcriptional regulation by specific *trans*-acting factors. Altogether these results demonstrate the throughput, flexibility and reproducibility of PTRE-seq.

## Results

### Design and application of PTRE-seq

**Fig. 1:**
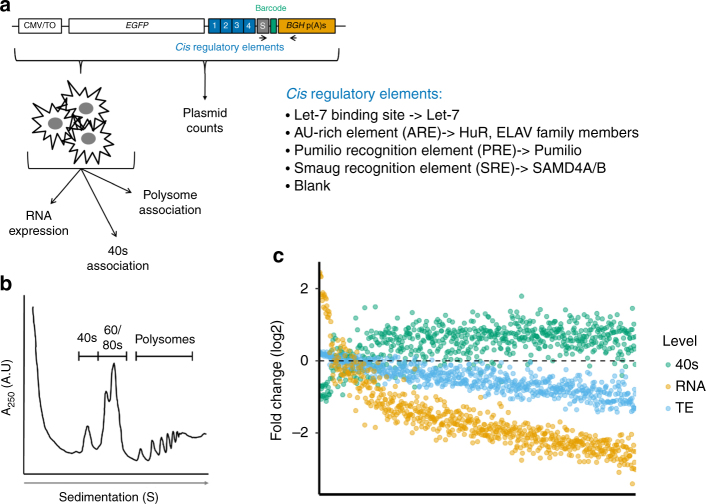
Design and application of PTRE-seq. **a** Schematic of the PTRE-seq library. Each *cis*-regulatory element (RE) within the library is inserted into an episomal reporter as shown. CMV/TO, cytomegalovirus promoter with the 5′UTR from the vector pCDNA5/FRT/TO. *EGFP*, enhanced green fluorescent protein. S, spacer sequence. *BGH* p(A)s, 3′UTR and polyadenylation signal from bovine growth hormone gene. Each unique synthetic 3′UTR, made up of binding sites for the REs shown, is represented by 10 barcodes. **b** Representative polysome profiling trace. mRNA was isolated from 40S, and polysome fractions. **c** Fold change of mRNA levels, translation efficiency, and 40S association for all reporters within the library. The reporters are arranged along the *x*-axis in decreasing order based on fold change

We developed PTRE-seq to quantify the individual and combined effects of RBP and miRNA binding sites in 3′UTRs. We created 642 unique synthetic 3′UTRs composed of combinations of a let-7 binding site, the Pumilio recognition element (PRE), the Smaug recognition element (SRE), AU-rich elements (ARE), and a control sequence (“blank”). Bioinformatic analyses indicate that nearly 300 human transcripts contain a PRE, miRNA-binding site and an ARE (Supplementary Fig. 1). Over 1900 transcripts contain a PRE and an ARE, 698 contain an ARE and a miRNA-binding site and 653 contain a PRE and a miRNA-binding site. Between 13 and 15% of each of these pairs of regulatory elements occur within 150 nt of each other, with many overlapping or immediately adjacent (Supplementary Fig. 1). We arranged the regulatory elements in four positions within the 3′UTR (Fig. 1a), resulting in 200 bp long regulatory element section. The library included all possible combinations of the five elements within the four positions to generate the 625-unique synthetic 3′UTRs. The remaining seventeen synthetic 3′UTRs in our library contained variants of let-7 binding sites. Every unique synthetic 3′UTR is present 10 times in the library, each time associated with a different co-transcribed barcode. These provide replicate measurements when the barcodes are used to quantify the relative abundance of reporter mRNAs in total or ribosome associated fractions from transfected cells. Barcoded synthetic 3′ UTR sequences were cloned downstream of a CMV promoter driven reporter gene to create a plasmid library.

We transfected HeLa cells with the library and collected the cells after 40 h. We isolated total RNA from a portion of the cells, and the remaining cells were lysed for polysome profiling to assay translational regulation. We collected mRNAs associated with the polysome fractions (translating ribosomes) and the 40S ribosome fractions (initiating ribosomes) (Fig. 1b and Supplementary Fig. 2). Messenger RNAs associated with the polysomal fractions are considered efficiently translated^48^. Since regulation of gene expression by various *cis* and *trans*-acting factors during mRNA translation often targets the translation initiation step, we separately analyzed the 40S fraction^49^. The 40S fraction contains mRNAs that are bound only by the small subunit of the ribosome during the translation initiation steps. We generated cDNA from the total RNA, polysome, and 40S associated mRNA and sequenced the barcodes to determine the relative abundance of every reporter in the library, in each fraction. Counts for every barcode in cDNA were normalized by counts determined by sequencing the input plasmid library. The Pearson correlation between replicate experiments ranged between 0.975 and 0.983 for total RNA, 0.703 and 0.787 for polysome associated RNA, and was 0.926 for 40S associated RNA (Supplementary Fig. 2), which allowed us to make quantitative comparisons between different synthetic 3′UTRs. To compute translation efficiency (TE), a measure of the reduction in translation beyond what is expected due to a reduction in mRNA levels, we normalized the barcode counts for each 3′UTR in the polysome fraction to its counts in total RNA. The same was done for the 40S associated RNAs to compute 40S association, which represents a proxy for the engagement of the translation initiation complex with mRNAs. In all cases, we determined the relative effect by normalizing to the control reporter, which contains four “blank” sequences in the synthetic 3′UTR. For most reporters, we observed both reduced RNA expression and reduced TE, which was concomitant with an increase in 40S association (Fig. 1c). Correlations can be seen between each of these metrics (Supplementary Fig. 2e–h). Summary statistics for PTRE-seq measurements of RNA expression and TE are shown in Supplementary Figure 3. We validated our PTRE-seq findings using quantitative PCR (qPCR) and fluorescence measurements of GFP for several individual reporters from the library (Supplementary Fig. 4). The data we have obtained using PTRE-seq reveal the ability of this method to capture evidence for post transcriptional regulation at different steps.

### Linear regression and thermodynamic modeling of results

The *cis*-elements in our library had strong effects in the data which we captured by fitting linear regression models for both RNA expression and TE to our data. For both the RNA expression model and the TE model, parameters included the identity of the element at each of the four positions and all pairwise interactions between elements at each position. The regression models captured the relationship between 3′UTR composition and relative RNA expression (five-fold cross-validation, Pearson correlation 0.87–0.93) (Supplementary Fig. 5) and the relationship between 3′UTR composition and TE (five-fold cross-validation, Pearson correlation 0.89–0.92) (Supplementary Fig. 6). The model predicted well the effects of individual elements and combinations of elements on RNA expression and TE (Supplementary Fig. 7). Interestingly, the models accurately predicted the RNA expression and TE of reporters containing three or four different binding sites using only the individual effect of each binding sites and pairwise interactions. Models fit with higher order interaction terms failed during cross-validation. This result, combined with the observation that models with individual effects and pairwise interactions perform well, suggests that higher-order interactions have, at most, only minimal effects on RNA expression and TE.

To gain mechanistic insights, we also fit a statistical thermodynamic model to our RNA expression and TE data^50,51^. Due to the position-dependent nature of ARE elements (described below), we excluded synthetic 3′UTRs containing ARE elements from this analysis. This model provides a formal biophysical framework to capture saturation effects and cooperative interactions between *cis*-acting elements. Each 3′UTR is described as a collection of states, in which each state represents a particular configuration of bound and unbound elements on a 3′UTR. The model uses parameters that describe the free energies of interaction between RBP/miRNA–RBP/miRNA and RBP/miRNA–mRNA to compute the probability, or weight of each state. These interactions can be neighboring or non-neighboring, however, our implementation of the model does not explicitly model position of the RBPs. In each state bound factors either facilitate or inhibit the recruitment of mRNA decay machinery (or the ribosome for TE), and the weights of the different states are used to compute the probability that the mRNA decay machinery (or the ribosome for TE) is present at an mRNA. In the model, this probability is proportional to the output RNA expression or TE^46,50^.

A thermodynamic model with four independent parameters, one each for the interaction of the decay machinery with either let-7, PRE, SRE, or the “blank” site, predicted observed TE (*R* = 0.92) and RNA expression well (*R* = 0.94). The good performance of these models suggests that let-7, PRE, and SRE function mostly independently on UTRs. In most cases adding interaction terms did not improve the fit of these models to the data. This observation suggests that some of the self-interaction terms in the linear regression models (described below) are likely due to saturation of binding on UTRs with high copy numbers of *cis*-acting sites. The thermodynamic model naturally accounts for saturation without the need for interaction terms and describes the situation when saturation causes additional sites to have little or no effect. In two cases, the thermodynamic model for TE did improve with the addition of interaction terms, one for interaction between adjacent let-7 sites and another for interaction between adjacent PRE and let-7 sites (*R* = 0.93, Supplementary Fig. 8), which suggests epistatic interactions between these elements that cannot be accounted for by binding site saturation. The thermodynamic model for RNA expression also improved with the addition of five interaction terms (*R* = 0.94, Supplementary Fig. 9). We sought to identify the trends in our data that underlie the strong performance of these models.

### PTRE-seq reveals mechanisms of *trans*-acting factors

**Fig. 2:**
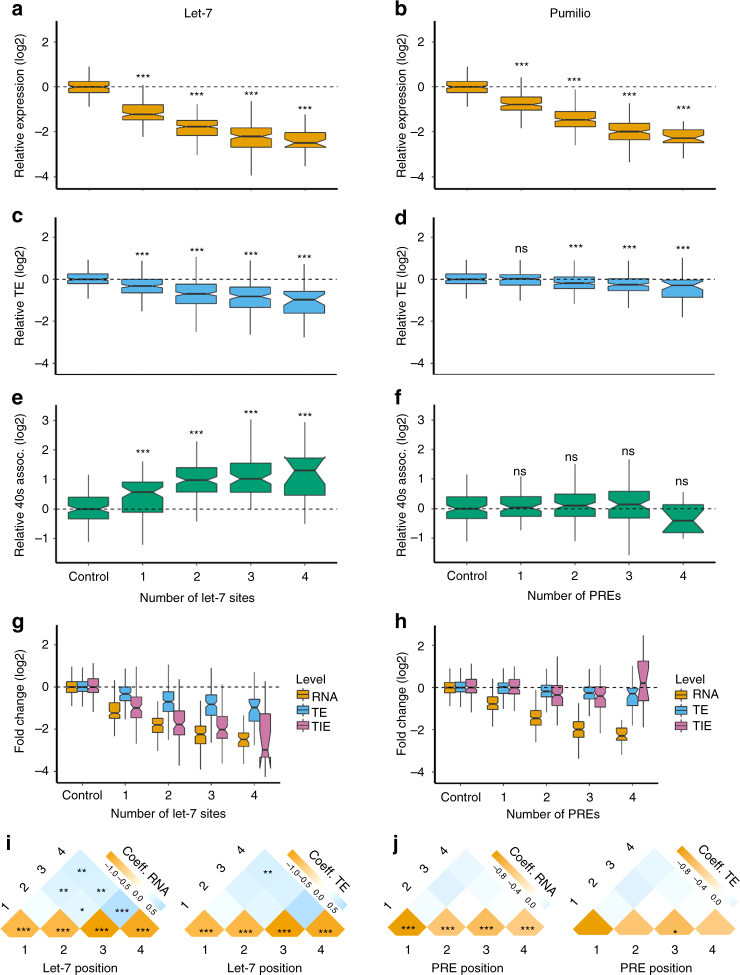
PTRE-seq reveals differences in the mechanism of repression by miRNAs and Pumilio Fold change of RNA (**a**) TE (**c**), and 40S association (**e**) of let-7 binding site containing reporters within the PTRE-seq library. Fold change of RNA (**b**) TE (**d**), and 40S association (**f**), of PRE containing reporters within the PTRE-seq library. For **a**–**d** *P < 0.05, **P < 0.01, ***P < 0.001, *t*-test with Bonferroni correction. For panels **a**–**f** the results for all constructs containing one, two, three, or four sites is shown. The data for each site in positions one-four are shown in Supplementary Fig. 9. Panels **g** and **h** show composite boxplots with fold change of RNA, TE, and translation initiation efficiency (TIE) for let-7 and PRE respectively. TIE was calculated by normalizing polysome associated RNA/40S associated RNA. **i** The regression coefficients for linear models with parameters corresponding to let-7 alone or in combination with other let-7 sites at positions 1–4, or **j**, PREs alone or in combination with PREs at positions 1–4. In **i** and **j**, the left panels show the coefficients for RNA while the right panels show the coefficients for TE. *P < 0.05, **P < 0.01, ***P < 0.001, *t*-test. Boxplot whiskers indicate the furthest datum that is 1.5*Q1 (upper) or 1.5*Q3 (lower). For clarity, outliers have been removed from boxplots but were used for statistical analysis

For each RNA element in our library there are a series of constructs that contain only that element and the control ‘blank’ sequence. This allowed us to study the individual and copy-number-dependent effect of each RNA element. For the let-7 binding site we observed a reduction in both relative RNA expression and TE (Fig. 2a, c and Supplementary Fig. 10a, c). This suggests that not only is the abundance of the RNA reduced by the addition of let-7 sites, but also that the remaining RNAs are translated poorly relative to the control message. Both effects were dependent on the number of let-7 binding sites in the synthetic 3′UTR and the effects appear to saturate with additional sites (Fig. 2a). While our linear regression model captures well the effects of individual let-7 sites, it is easily influenced by saturation effects and thus cannot distinguish between saturation effects and true epistatic interactions (Fig. 2i). To counter this we employed our thermodynamic model. The thermodynamic model requires an interaction term between let-7 binding sites that stabilizes RNA for a good fit (Supplementary Fig. 9). Since the thermodynamic model is robust to saturation effects, this interaction term suggests epistatic antagonism between let-7 binding sites.

The Pumilio recognition element (PRE) behaved differently than the let-7 binding site. Like the let-7 binding site, PREs also decreased RNA expression, but had a much more modest effect on TE (Fig. 2b, d and Supplementary Fig. 11b, d). Additional PREs appeared to show a modest saturation effect on RNA expression (Fig. 2b). However, our thermodynamic model for RNA or TE revealed no significant epistatic interactions between PREs and therefore we have excluded this term from the model. While both the let-7 binding site and PREs reduced TE, only the let-7 binding site affected the association with the 40S ribosomal subunit (Fig. 2e, f). Let-7 binding sites increased 40S association in a manner dependent on the number of sites. This could be caused by slowed scanning of the 5′UTR or recruitment of the large subunit of the ribosome. These data show a clear distinction between the mechanisms of post-transcriptional regulation by Pumilio and the miRISC. These differences are illustrated by normalizing polysome associated RNA counts by 40S associated RNA counts, which produces a value we term translation initiation efficiency (TIE). TIE is reduced substantially, and in a copy-number-dependent manner by let-7 and only modestly by Pumilio (Fig. 2g, h). Taken together our results suggest that Pumilio works mostly by destabilizing its target mRNAs, while the miRISC functions both by affecting mRNA stability and by inhibiting translation initiation. This mechanistic difference was clearly captured by PTRE-seq.

### PTRE-seq unveils the effect of miRNA-target base-pairing

**Fig. 3:**
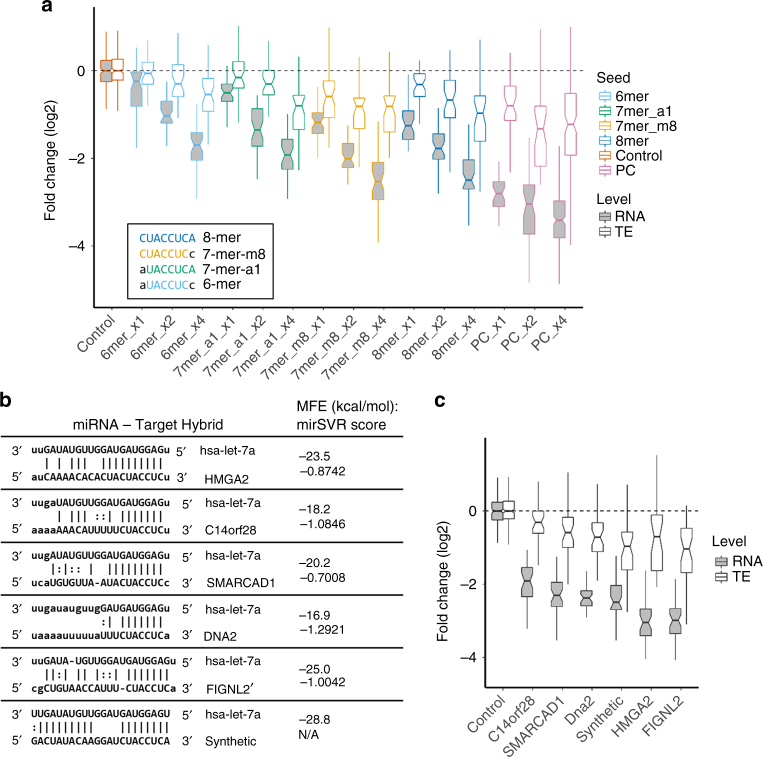
PTRE-seq reveals the effect of the let-7 binding site on repression. **a** Comparison of the fold change of reporters containing synthetic let-7 binding sites with altered seed binding. Also shown are reporters containing let-7 binding sites that have perfect complement (PC) binding to let-7. Each seed binding variant is present in either one, two or four copies. The inset describes the seed binding region of each seed-binding variant site. **b** Table describing the natural and synthetic let-7 binding sites used in this study. MFE, minimal free energy^71^. mirSVR, mirSVR score^72^. **c** Fold change of RNA and TE for reporters containing four copies each of natural or synthetic let-7 binding sites. Boxplot whiskers indicate the furthest datum that is 1.5*Q1 (upper) or 1.5*Q3 (lower). For clarity, outliers have been removed from boxplots but were used for statistical analysis

The efficiency of miRNA-mediated repression depends on the number and quality of binding sites in its target^34,35,52,53^. Nucleotides 2–7 of the miRNA constitute the “seed” sequence, and weak seed pairing reduces the effectiveness of miRNA-mediated repression^34,35,52,53^. In addition to the 625 combinations of regulatory elements described above, we included constructs in the library to study the effect of base-pairing between the miRNA and its target on repression. This included a series of constructs with one, two, or four binding sites for let-7 with either 6-mer, 7-mer-a1, 7-mer-m8 or 8-mer base-pairing in the seed region^52^, as well as binding sites for let-7 that have perfect base-pairing with the target. We observed a clear copy-number-dependent and seed-pairing dependent effect on RNA expression and TE for these reporters (Fig. 3a, Supplementary Figure 12). The repression at the level of RNA and TE was greatest for target sites with 8-mer or 7-mer-m8 pairing. A single copy of the perfect complement let-7 binding site was more effective at reducing RNA expression than four copies of the binding site with a mispairing bulge (Fig. 3a). In addition to studying the effect of seed-pairing alone, we also studied the effect of endogenous let-7 binding sites. For this we made constructs containing four copies of a let-7 binding site from the 3′UTR for *HMGA2*, *SMARCAD1*, *DNA2*, *C14orf28* and *FIGNL2*. While, the synthetic binding site is predicted to have the most favorable binding (Fig. 3b), the sequences from two of the natural 3′UTRs (*HMGA2* and *FIGNL2*) reduced RNA expression to a greater extent (Fig. 3c). We suspect that secondary structure around the let-7 binding sites in these reporters is contributing to let-7 binding. This can be seen by making a simple linear regression model for fold change of RNA expression with base-pairing minimal free energy (MFE) and 3′UTR secondary structure MFE as parameters. A model that includes each parameter and an interaction term gave a better fit (*R* = 0.81) than base-pairing MFE (*R* = 0.56) or secondary structure MFE (*R* = 0.12) alone. Because this model was made with only a few data points it is only suggestive. This secondary structure of the 3′UTR could explain the observation that some binding sites, even with better thermodynamics, were not as well repressed.

### Pumilio does not enhance miRISC function

**Fig. 4:**
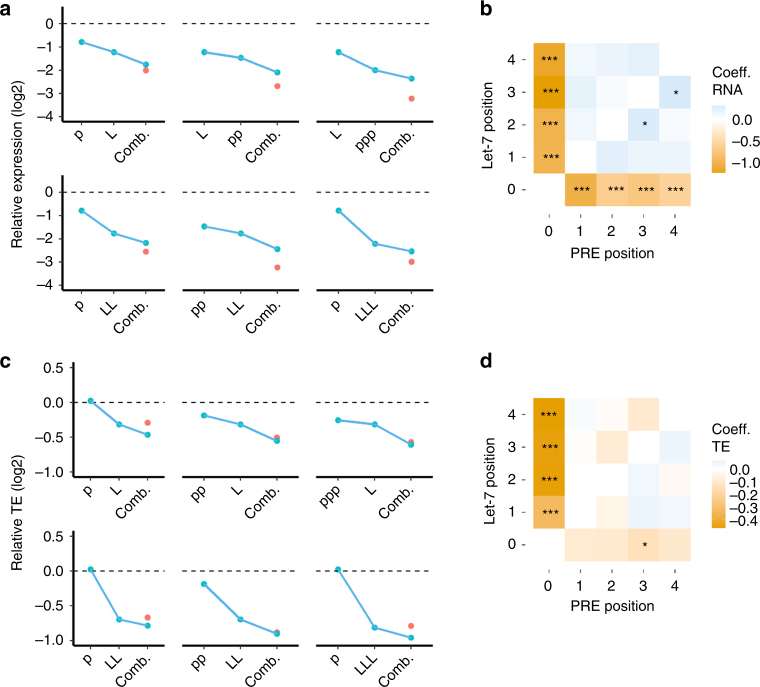
Pumilio and miRNAs function independently. **a** The effect of a let-7, PRE or a combination of the two elements on relative expression, and **c** relative TE. The median relative expression or TE is plotted across all barcodes and replicates. Red dot, the product of each individual effect, the expected result assuming independence. The regression coefficients from the linear regression model for RNA expression (**b**), and TE (**d**), for the parameters corresponding to let-7 or PREs alone or interactions between positions containing let-7 or PREs. For **a** and **c** L = let-7 binding site, p = PRE, *P < 0.05, **P < 0.01, ***P < 0.001, *t*-test

Enhancement of miRNA-mediated repression by the RBP Pumilio has been observed for a handful of targeted mRNAs^29,30^. In our library, the combination of a let-7 binding site and a PRE resulted in a reduction of RNA expression that was slightly less than the product of their individual effects (Fig. 4a). This was true for every combination we tested. The coefficients from our linear regression model for RNA levels are positive for all combinations of PRE and let-7, while the coefficients for each alone is negative (Fig. 4b). For TE we observed a modest enhancement of repression for some combinations and no effect for others (Fig. 4c), this was captured by our linear regression model which showed a mix of positive and negative coefficients for the combinations of PRE and let-7 (Fig. 4d). The pairwise arrangement of let-7 binding sites and PREs had no effect on repression (Supplementary Fig. 12). These data suggest a slight antagonism between the two elements in regard to their effects on RNA stability, and is reminiscent of the saturation we observed with additional let-7 or Pumilio binding sites. The thermodynamic model for TE includes a statistically non-significant anti-cooperative interaction between let-7 and PRE sites, while the model for RNA decay includes anti-cooperative interaction terms for a subset of let-7 and PRE binding site combinations (Supplementary Fig. 8 and 9). Thus, the miRISC and Pumilio function independently in most UTRs and reduced repression seen with combinations of sites is mostly because of saturation effects. As miRNAs and Pumilio are thought to promote mRNA decay using the same pathway it is not surprising that when both are bound to the same message there is no enhanced degradation^6–8,16,17^.

### Position dependent effects of AU-rich elements

**Fig. 5:**
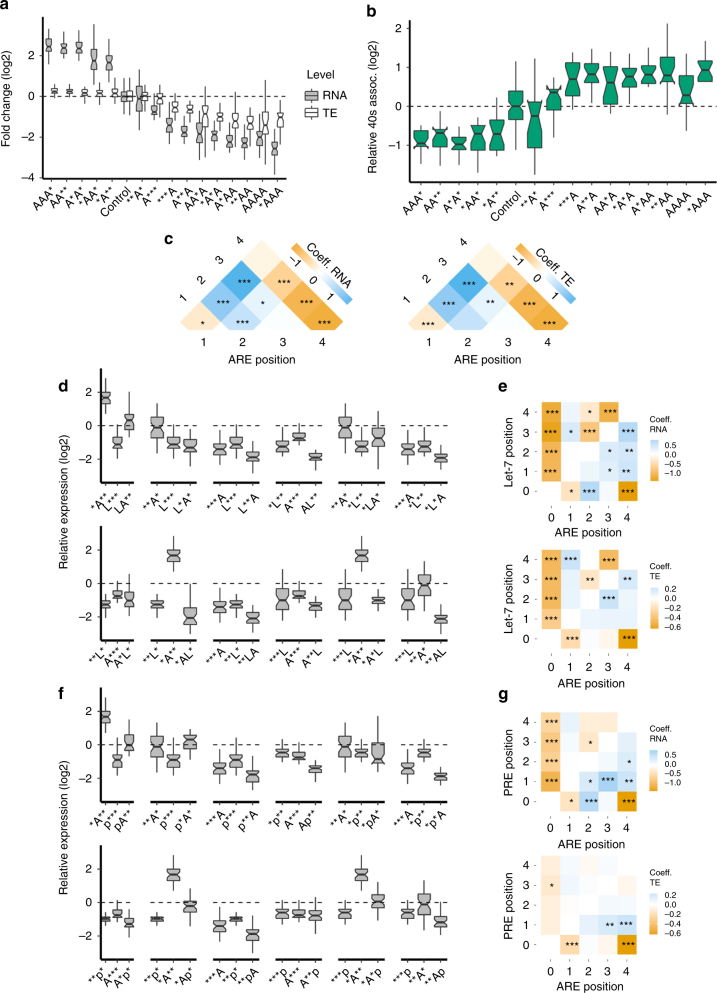
AU-rich elements modulate repression by Pumilio and miRNAs. **a** The position of an ARE within the synthetic 3′UTR determines the relative TE or RNA expression. **b** The relative 40s association of ARE containing reporters. **c** Heatmap of the regression coefficients for the parameters corresponding to AREs alone. Left panel shows coefficients for RNA expression and the right panel shows coefficients for TE. **d** AREs modulate repression by miRNAs in a position-dependent manner. The green box highlights an example of stimulation of miRNA-mediated RNA destabilization by an ARE. **e** The regression coefficients for the parameters corresponding to let-7 or AREs alone or interactions between positions containing let-7 or ARE. **f** AREs modulate repression by PREs in a position dependent manner. **g** The regression coefficients for the parameters corresponding to PREs or AREs alone or interactions between positions containing PRE or ARE. For **a**, **b**, **d** and **f**, * = Blank, A = ARE, L = let-7 and p = PRE. For **c**, **e** and **g**, *P < 0.05, **P < 0.01, ***P < 0.001, *t*-test. Boxplot whiskers indicate the furthest datum that is 1.5*Q1 (upper) or 1.5*Q3 (lower). For clarity, outliers have been removed from boxplots but were used for statistical analysis

Several RBPs can bind AU-rich elements. ARE binding proteins, such as HuR (ELAVL1), can either stabilize or destabilize target mRNAs, and can enhance or repress translation^22^. Other RBPs, such as tristetraprolin (TTP) and AUF1 (hnRNPD), are also ARE-binding proteins (ARE-BP)^22^. In our library AREs either enhanced or repressed RNA expression and TE depending on their position in the 3′UTR (Fig. 5a). In the first or fourth position in our synthetic 3′UTR, ARE reduces both RNA expression and TE, while an ARE in the second position increases RNA expression and TE, and an ARE in the third position has no effect on either metric. The combination of multiple AREs altered this position-dependent effect. Generally, any combination with an ARE in position four had reduced RNA and TE while all other combinations had increased RNA and TE. We observed similar effects on 40S association where any combination of ARE that reduced TE resulted in increased 40S association and vice versa for those that increased TE (Fig. 5b). These observations were captured by our linear regression model for RNA expression, which showed an ARE at position four to have a negative coefficient and an ARE at position two to have a positive coefficient (Fig. 5c). Since the linear regression model cannot distinguish between saturation effects and epistatic interactions it is difficult to assign a cause. However, the results clearly show instances were AREs in specific arrangements lead to increased or decreased RNA expression and/or TE.

### AU-rich elements modulate activity of miRNAs and Pumilio

The AU-rich element binding protein HuR has been shown to both activate and inhibit miRNA-mediated repression^2^. During recovery from stress HuR relieves miRNA-mediated repression of the catalase mRNA (*Cat1*)^32^. In contrast, HuR binding to the *c-Myc* 3′UTR activates miRNA-mediated repression^31^. We observed AREs in our library either enhancing or suppressing miRNA-mediated repression in a position dependent manner (Fig. 5d and Supplementary Fig. 13). For example, a let-7 binding site at position three reduces RNA expression while an ARE at position two increases RNA expression, but the combination of ARE and let-7 reduces RNA expression more than the let-7 binding site alone. Our linear regression likely captured some of these effects but because it cannot distinguish between saturation effects and epistatic interactions we cannot assign a cause (Fig. 5e). However, it is clear that in some cases specific combinations of let-7 binding sites and AREs resulted in obvious changes to RNA expression or TE, for instance changing from a message that is stable to one that is unstable, see above example. We observed similar position-dependent modulation of Pumilio activity by AREs (Fig. 5f and g). Together these data show that AU-rich element binding proteins can modulate the repression by the miRISC and Pumilio in a position-dependent manner, even though, as shown above, Pumilio and let-7 utilize different mechanisms to repress RNA expression and TE.

### Post-transcriptional regulation varies across cell types

**Fig. 6:**
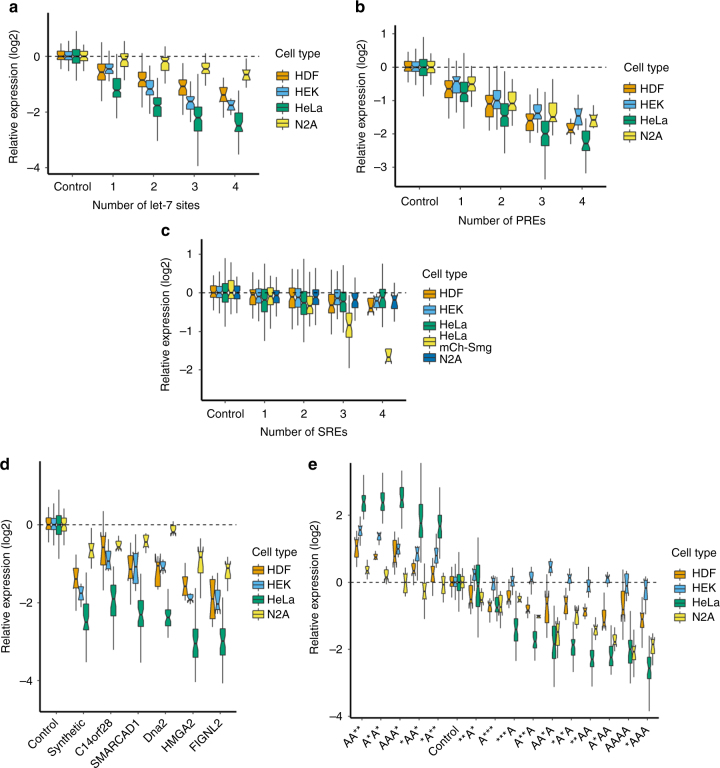
The regulatory capacity of miRNAs and AU-rich elements vary across cell types The relative expression of reporters containing let-7 binding sites (**a**), PREs (**b**), SREs (**c**), natural binding sites for let-7 (**d**), or AREs (**e**). In panel **c** HeLa-mCh-Smg refers to HeLa cells that were cotransfected with the PTRE-seq library and a plasmid for expression of mCherry-Smaug. HDF, neonatal human dermal fibroblasts. HEK, human embryonic kidney. N2A, mouse neuro2A. For **e** *=Blank and A=ARE. Boxplot whiskers indicate the furthest datum that is 1.5*Q1 (upper) or 1.5*Q3 (lower). For clarity, outliers have been removed from boxplots but were used for statistical analysis

In addition to HeLa cells, we also transfected our PTRE-seq library into three other cells types: human embryonic kidney (HEK293), human neonatal dermal fibroblast (HDF), and a mouse neuroblastoma (N2A). We observed wide variation in the effect of each regulatory element tested across the four cell lines. It is possible that some of these changes could be caused by differences in transfection efficiency or transcription across the cell lines tested. The let-7 binding site caused robust reduction of RNA expression in HeLa cells, but this effect was smaller in magnitude in HEK293, HDF and N2A (Fig. 6a, d and Supplementary Fig. 14). Neuroblastoma cells are thought to have very little expression of let-7^54^. In contrast, we observed modest variations in the magnitude of repression by PREs across the four cell lines (Fig. 6b). PREs were most effective in HeLa and least effective in N2A or HEK293 cells. Interestingly, we observed only a very modest reduction in RNA expression and no effect on TE (Supplementary Fig. 15) for reporters containing SREs across all cell-lines. Only when we overexpressed the *Drosophila* homolog of SAMD4A and SAMD4B, Smaug (mCh-Smg) did we see a substantial reduction in RNA expression (Fig. 6c). For AREs, the cell type not only altered the magnitude of the effect but could also abrogate the effect entirely (Fig. 6e). For example, in HeLa the AREs could both reduce or increase RNA expression, while in HEK293 we only observed increased RNA expression by AREs. Conversely, in N2A we observed robust reductions in RNA expression by AREs but very modest increases in RNA expression. As AREs are known to be bound by multiple RBPs this finding suggests the presence of a different profile of active ARE-binding RBPs in each cell type.

## Discussion

To better understand the post-transcriptional regulation of mRNAs, we must determine how regulatory factors function both independently and in combination with each other. Towards this end we developed PTRE-seq, a powerful new high-throughput tool for interrogating the additive and combined effects of binding sites for RBPs and miRNAs on RNA stability and translation. As PTRE-seq is extended to additional RBPs and miRNAs, we will better understand the network of molecular interactions that comprise post-transcriptional regulatory systems.

Using PTRE-seq we observed decreased RNA levels and decreased association with polysomes mediated by the let-7 miRNA. By fractionating the cell lysates before analysis, we determined that the reduction in polysome associated RNA was more than could be accounted for by the decrease in RNA levels alone, indicating that let-7 reduces RNA levels and reduces the efficiency with which the remaining RNA is translated. This decrease in translational efficiency also correlated with an increase in 40S association of mRNAs targeted by the miRNA let-7. These results are consistent with a proposed model in which the miRISC inhibits translation initiation at the scanning step by induced dissociation of the helicase subunit eIF4A of the eIF4F complex^3–5,9,55^. The reduced rate of scanning increases the time that the 40S ribosome is bound to the message prior to identification of the start codon and recruitment of the 60S ribosome. This delay in subunit joining would increase the time mRNAs spend bound by the 40S ribosome while reducing translation efficiency. The ability of PTRE-seq to separate effects on RNA levels from effects on different steps of translation is an important advantage of this method.

Beyond identifying the mechanism of miRNA-mediated repression another major challenge in the field remains defining binding sites with gene regulatory activity of the thousands of miRNAs in the cell^56–60^. Using PTRE-seq we were able to study the efficacy of target sites for let-7. Our results are consistent with previous studies: miRNA efficacy depends on thermodynamics of binding and 3′UTR structure^34,35,52^. We observed stronger repression for messages containing more base-pairing within the seed sequence: 8-mer >7-mer >6-mer. This finding was consistent with previous studies of endogenous miRNA targets^34,35^. Furthermore, reporters containing four copies each of endogenous let-7 binding sites showed variable repression that was not always dependent on thermodynamics of base-pairing. A simple linear regression model revealed that the secondary structure around the let-7 binding sites contributed to the magnitude of repression. This finding is consistent with a model for miRNA target prediction which incorporates the thermodynamics of miRNA binding and secondary structure near the binding site^56^. This type of analysis could be used for other miRNAs to empirically define their binding sites with largest impact on gene regulation.

In contrast to let-7, Pumilio decreased RNA levels with only very modest effects on polysome association and no effect on 40S subunit binding. Our results are consistent with the findings that Pumilio and miRNAs inhibit translation at different steps^3–5,9,16,19^. It is also possible that the differences we observed between let-7 binding sites and PREs could reflect differences in the kinetics of repression by the miRISC or Pumilio. Besides their individual roles in regulation of gene expression, miRNAs and Pumilio have been shown to function together. In some cases, Pumilio can activate miRNA-mediated repression of specific mRNAs^29,30^. However, in our experiments the effects of let-7 and Pumilio were largely independent. Pumilio may only activate particular miRNA targets by opening certain secondary structures and enabling miRNA binding^30^. To test this model PTRE-seq could be performed on synthetic messages carrying miRNA binding sites and PREs in the context of varying secondary structures.

The effects of let-7 or Pumilio sites showed almost no dependency on their position in the 3′UTR. In contrast, we observed a strong positional effect for AREs. AREs are bound by several RBPs including the ELAVLs, Auf1, and TTP, and different ARE binding proteins can stabilize or destabilize mRNA targets, as well as repress or enhance translation. The dependency of ARE on position could be explained if different ARE binding proteins are binding at different positions in the 3′UTR. Although the sequence of the ARE is the same at each position, the flanking sequence context is different, and RNA secondary structure may vary based on the position of the ARE (Supplementary Fig. 16). This altered structure might affect which ARE-BP bind to the sequence. The varied effects of AREs across cell-lines are consistent with this hypothesis. While AREs both increased and decreased RNA expression in most cell lines tested, in HEK293 we only observed increased RNA expression. As the expression of ARE-BPs is known to vary across cell and tissue types, this finding suggests that the ARE-BPs with different effects are binding to the same reporters in different cell-lines^22^. In any given cell-line, the cumulative effect of multiple ARE-BPs determines the overall activity of AREs.

Our experiments revealed strong epistatic interactions between AREs and sites for let-7 and Pumilio. The AREs either enhanced or suppressed miRNA- and Pumilio-mediated repression, depending on their position in the 3′UTR. These effects were not dependent on the proximity in linear sequence space between the two binding sites. These results demonstrate that AREs can modulate repression by both miRISC and RBPs such as Pumilio. Alternatively, the presence of the PRE or miRNA binding site may modulate the effect of the ARE by changing the secondary structure of the mRNA. These observations warrant future studies into which ARE-BP are responsible for these epistatic interactions, and whether the effects are mediated through mRNA secondary structure or potentially through interactions between *trans*-acting factors. An intriguing possibility is that the ARE may be bound by cytoplasmic polyadenylation element binding protein, CPEB. The consensus binding site for CPEB is UUUUUAU^61^, but CPEB also binds the sequence UUUUAU^62^, which appears once in the ARE used in our library. CPEB has been previously shown to work with Pumilio in regulating mRNA translation^63^.

While we observed robust effects on the RNA expression and translation of reporters containing let-7 binding sites, PREs and AREs, in our experiments, the SRE caused only modest changes in RNA expression and no change in TE or 40S association. When we overexpressed *Drosophila* Smaug in HeLa cells, we observed a reduction in the RNA levels of SRE containing reporters. This suggests that at least in the cell-lines we tested the mammalian Smaug homologs, *SAMD4A* and *SAMD4B*, are expressed at low levels or are not efficacious.

Our results provide further evidence for the mechanisms of post-transcriptional regulation by miRNAs, Pumilio and AREs. PTRE-seq will serve as a valuable tool studying the effects of multiple *cis*-acting elements, both individually and in combination, and for unraveling their effects on different aspects of RNA stability and translational control.

## Methods

### Construction of library

To create the PTRE-seq library we first generated the plasmid pCDNA5/FRT/TO-EGFP-RE. EGFP was PCR amplified with EGFP-F and RE-R primers, Supplementary Table 1. This appended a single *NheI*, *EcoRV*, and *KpnI* sites downstream of EGFP. The PCR product was ligated into pCDNA5/FRT/TO that had been previously cut with *PmeI*.

A pool of 6500 unique 200-mer oligonucleotides was ordered from Agilent Technologies™. Oligonucleotides were designed to contain all combinations of either a let-7 binding site, PRE, SRE, AU-rich element or a “blank” control sequence. The sequence for each of these elements is described in Supplementary Table 2. Each of these unique combinations was synthesized with 10 different 9 bp barcodes. This provided a total of 6250 oligonucleotides. The remaining oligonucleotides consisted of 40 additional copies of the control sequence (4 place holders, “blanks”), 50 copies of a low expression control (4×let-7 perfect complement) and a series of constructs containing natural or synthetic let-7 sites. In total, the library consisted of 642 unique ‘synthetic 3′UTRs’ each with 10 unique barcodes, except for the controls described above. The sequences of each of these “synthetic 3′UTRs” are in the Supplementary Data 1. Each oligo has a 5′ and 3′ priming region which are identical across all oligonucleotides. The oligonucleotides also contained a restriction enzyme sites for subsequent cloning. A generic oligonucleotide appears as follows: 5′ – GTAGCATCTGTCCGCTAGC-132nt regulatory element-ATGCATcGATATCaCTCGAGxxxxxxxxxGGTACCCGACTACTACTACG – 3′. The restriction enzymes are underlined and are from 5′ to 3′: *NheI*, *NsiI*, *EcoRV*, *XhoI*, and *KpnI*.

The library was PCR amplified for four cycles using Phusion High-Fidelity polymerase (NEB) and primers Lib_F and Lib_R. We cloned the amplicon into pCDNA5/FRT/TO-EGFP-RE using *NheI* and *KpnI*. We prepared plasmid DNA from ~40,000 colonies to generate library RE_Array*. We then cloned a “spacer” sequence in between the regulatory elements and the barcode. This “spacer” is the reverse of a sequence within the BGH 3′UTR and is used for amplification of the barcodes from cDNA or plasmid. The “spacer” was ordered as a pair of oligonucleotides that were annealed to form a dsDNA oligonucleotide with overhangs compatible with DNA cleaved by *NsiI* and *XhoI*. The “spacer” was cloned into the RE_Array* using *NsiI* and *XhoI*. We collected plasmid DNA from ~250,000 colonies to generate the library RE_Array_1.

To clone individual reporters from the library we sequenced 94 colonies from the RE_Array* library. We chose from those clones seven reporters of interest. For the control reporter and three reporters targeted by let-7 (*7**, 7777, and 7pc-x2), we ordered oligonucleotides that were ligated into the vector pCDNA5/FRT/TO-EGFP-RE as described above. The “spacer” was ligated into the plasmids containing the reporters, as described above.

### Cell culture and transfection

HeLa (CCL-2.2, ATCC), HDFn (C0045C, Thermo Fisher), N2A (CCL-131, ATCC) and T-REx^TM^-293 cells (R71007, Thermo Fisher) were grown in DMEM (Gibco) supplemented with 10% heat-inactivated FBS (Gibco), 1×Penicillin streptomycin and glutamine (Gibco) and 1 × MEM Non-Essential Amino Acids (Gibco). Transfection was carried using the Neon Transfection System (Invitrogen) per manufacturer protocol. For each transfection, 2.5 × 10^6^ cells were electroporated with 8 µg of RE_Array_1. For transfection of mCh-Smg, we electroporated 8 µg of pCDNA-D40-mCh-Smg along with 8 µg of RE_Array_1 into HeLa cells as described above. The mCh-Smg plasmid was made by PCR amplifying the Smaug coding sequence (CDS) from *Drosophila* S2 cell cDNA using the primers described in Supplementary Table 1. The Smaug CDS was fused to mCherry through overlap PCR using primers described in Supplementary Table 1. This PCR product was cloned into pENTR-D-TOPO (Invitrogen) and subsequently recombined into pcDNA-D40 (Invitrogen) using LR Clonase (Invitrogen).

For transfection of the individual reporters we used Effectene (Promega). The cells were transfected in a 12-well plate with 500 ng each of the EGFP reporter and pCDNA-mCherry^64^ per the manufacturers protocol. The cells were split 24 h later into two separate 12-well plates and a 96-well plate. Forty hours after transfection the fluorescence was measured using a Synergy H4 plate reader (BioTek), at the same time RNA and protein was isolated from the 12-well plates.

### RNA isolation and polysome profiling

Total RNA was isolated using Qiagen RNA mini-prep per manufacturer’s protocol. For HeLa cells we collected total RNA from four biological replicates, when testing the library in other cell types we collected one biological replicate. For polysome profiling, cells were treated with 10 µg ml^−1^ cycloheximide for 5 min prior to harvesting and counting. A total of 3 × 10^6^ cells were lysed and the lysate was subjected to ribosome fractionation using 7 to 47% sucrose gradient (Teledyne ISCO) as described previously^65^. In brief, cells were treated with 10 µg/ml cycloheximide for 5 min prior to harvesting and counting. A total of 3 × 106 cells were lysed in 250 µL of polysome lysis buffer (20 mM Tris pH 7.2, 130 mM KCl, 10 mM MgCl2, 2.5 mM DTT, 0.5% NP-40, 0.2 mg/ml Heparin, 0.5% Sodium Deoxycholate, 10 µg/mL cycloheximide and 200 U/mL RNase Inhibitor) on ice for 20 min prior to clearing at 8000 × *g* for 10 min at 4 °C. The lysate was layered over a 7–47% sucrose gradient and subjected to centrifugation at 160,000 × *g* for 3 h at 4 °C. The gradient was fractionated by upward displacement with constant measurement of absorbance at 254 nm using a Teledyne ISCO fractionator. RNA was isolated from 40S and polysome fractions using Ribozol (Amresco). We collected polysome fractions from four biological replicates and 40S fractions from two. Isolated RNA was treated with Turbo DNase (Ambion). For qPCR of rRNA from total, 40S and polysome fractions, first strand cDNA synthesis was carried out using Superscript IV reverse transcriptase (Invitrogen) with random hexamer priming. qPCR was performed with iQ^TM^ SYBR Green master mix with the 18S and 28S rRNA primers described in Supplementary Table 1.

For qPCR of the individual reporters: RNA was isolated using the Qiagen RNA mini-prep per manufacturer protocol. Isolated RNA was treated with Turbo DNase (Ambion) prior to first strand cDNA synthesis using Superscript Vilo (Invitrogen). Quantitative PCR was performed with EGFP and mCherry primers, Supplementary Table 1.

### Illumina library preparation

First strand cDNA synthesis for ribosome associated RNA or total RNA was carried out using Superscript IV reverse transcriptase (Invitrogen) with random hexamer priming. The barcode was amplified from cDNA or plasmid using RE_Amp_F and RE_Amp_R primers with Phusion-HF MM (NEB): 98 °C for 1 min, 22 cycles: 98 °C for 10 s, 55 °C for 30 s, 72 °C for 30 s, and 72 °C for 5 min. The amplicon was purified using Nucleospin Gel and PCR cleanup kit (Macherey Nagel) and subsequently digested with *XhoI* and *SpeI*. The digestion product was purified as before and ligated to the Illumina adapters described in Supplementary Table 1. This product was amplified using Il_Enrich_F and Il_Enrich_R with Phusion HF MM (NEB): 98 °C for 1 min, 21 cycles: 98 °C for 10 s, 66 °C for 30 s, 72 °C for 30 s, and 72 °C for 5 min. This product was resolved by agarose gel electrophoresis and the appropriate sized band was excised and purified using Nucleospin Gel and PCR cleanup kit (Macherey Nagel).

The Illumina library was multiplexed and run on four lanes of Illumina NextSeq machine. Barcodes counts were determined. Only barcodes with greater than >10 counts in the cDNA and plasmid pools were used for analysis.

### Western blot analysis

Cells that were transfected with individual reporters were lysed with Lysis Buffer (50 mM Tris pH 7.8, 150 mM NaCl, 1% NP-40). Western blot analysis was performed, as described previously^64^. The following primary antibodies were used in western analysis at the given dilution: GFP, 1:2000 (Clontech, 632381); β-actin-HRP, 1:2000, (Cell Signaling, 12262); Anti-mouse IgG HRP, 1:10,000 (Cell Signaling, 7076S).

### Data analysis

Relative RNA expression for each regulatory element was calculated as described below. In brief cDNA counts for each barcode were normalized by the plasmid counts for the same barcode. The normalized expression was set relative to the median normalized expression of the control, 4×Blank.

$${\mathrm{Relative}}\;{{\rm RNA}}\;{{\rm Expression}} = \log _2\left[ {\frac{{\frac{{{\rm cDNA}_x}}{{{\rm plasmid}_x}}}}{{{\rm median}\left( {\frac{{{{\rm cDNA}_{{\rm control}}}}}{{{{\rm plasmid}_{{\rm control}}}}}} \right)}}} \right]$$

Relative TE for each regulatory element was calculated as described below. In brief polysome associated cDNA (pRNA) counts for each barcode were normalized by the plasmid counts for the same barcode. The normalized expression was set relative to the median normalized TE of the control, 4×Blank.

$${\mathrm{Relative}}\;{\mathrm{TE}} = \log _2\left[ {\frac{{{\mathrm{pRNA}}_{x}{{\mathrm{/}}}{\mathrm{cDNA}}_x}}{{{\rm median}\left( {\frac{{{{\rm pRNA}_{{\rm control}}}}}{{{{\rm cDNA}_{{\rm control}}}}}} \right)}}} \right]$$

Relative 40S association for each regulatory element was calculated as described below. In brief, 40S associated cDNA (srRNA) counts for each barcode were normalized by the plasmid counts for the same barcode. The normalized expression was set relative to the median normalized 40S association of the control, 4×Blank.

$${\mathrm{Relative}}\;40{\mathrm{s}}\;{\mathrm{Association}} = \log _2\left[ {\frac{{{\mathrm{srRNA}}_x{{\mathrm{/cDNA}}}_x}}{{\mathrm{median}\left( {\frac{{{{\rm srRNA}_{{\rm control}}}}}{{{{\rm cDNA}_{{\rm control}}}}}} \right)}}} \right]$$

Relative TIE for each regulatory element was calculated as described below. In brief, cDNA from polysome associated RNA (pRNA) counts for each barcode were normalized by the cDNA from 40S associated RNA (srRNA) The normalized expression was set relative to the median normalized TIE of the control, 4 × Blank.

$${\mathrm{Relative}}\;{\mathrm{TIE}} = \log _2\left[ {\frac{{{\mathrm{pRNA}}_x{{\mathrm{/srRNA}}}_x}}{{{\rm median}\left( {\frac{{{{\rm pRNA}_{{\rm control}}}}}{{{{\rm srRNA}_{{\rm control}}}}}} \right)}}} \right]$$

### Linear regression model

For each synthetic 3′UTR, we calculate median fold change across all 10 barcodes. Median fold change values are fit to linear model with interacting terms for the let-7 binding site, PRE, SRE, AU-rich element or space-holding sequence, at four positions using the lm function in R^66,67^. Coefficients are obtained in reference to “blank” sequence at each position. For cross-validation, we randomly divide the data into five parts and use 80% of the data to train and tested on the remaining 20%. This procedure is repeated five times. The parameters for our linear regression model are shown below:

$${\rm Relative}\;{\rm RNA}\;{\rm Expression}\sim {\mathrm{P}}_1 + {\mathrm{P}}_2 + {\mathrm{P}}_3 + {\mathrm{P}}_4 + {\mathrm{sum}}\left( {{\mathrm{P}}_{{i}} \ast {\mathrm{P}}_{{j}}} \right)$$

$${\rm Relative}\;{\rm TE}\sim {\mathrm{P}}_1 + {\mathrm{P}}_2 + {\mathrm{P}}_3 + {\mathrm{P}}_4 + {\mathrm{sum}}\left( {{\mathrm{P}}_{{i}} \ast {\mathrm{P}}_{{j}}} \right)$$

where *j* = 1 to 4 and *i* ≠ *j*, * = Interactions

### Thermodynamic model

To model reporter mRNA levels as a function of the factors bound to their synthetic 3′UTRs we implemented a modified version of a thermodynamic model of transcriptional regulation proposed previously^68^ and then later modified^45,46,51^. We modified this framework slightly to model post-transcriptional regulation of mRNA by RNA binding proteins and miRNAs as described below.

In our model we specify that each 3′UTR has four binding sites for factors that bind RNA (RNA binding proteins and miRNAs). When a spacer sequence is present at one of the sites we assume that no factor is bound to the site. We also specify that the 3′UTR has one additional binding site for a rate-limiting factor that controls the regulation of the mRNA. RBPs and miRNAs bound on the 3′UTR either facilitate or inhibit the binding of this rate-limiting factor. The primary assumption of this model is that the equilibrium binding of RBPs and miRNAs determine the occupancy of the rate-limiting factor on the 3′UTR. The equilibrium assumption is justified because the timescales at which RBPs and miRNA bind and unbind their sites are much faster than the timescale at which regulation of mRNA levels occur^69,70^.

In our model of synthetic 3′UTRs we specify that there are five (*N*) total binding sites, four for RBPs and miRNAs, and one for the rate-limiting regulator. Each 3′UTR can exist in many possible states, where a state is a particular configuration of bound factors. A state of the 3′UTR is specified by giving the occupancy *σ*_*i*_, either 0 or 1, of each site. The model calculates a Boltzmann weight for each state of the 3′UTR using:

$$W\left( {\sigma _1, \ldots ,\sigma _N,\sigma _{1,1}, \ldots ,\sigma _{N,N}} \right) = {\mathrm{exp}}\left( { - \mathop {\sum }\limits_{i = 1}^N q_i\sigma _i - \mathop {\sum }\limits_{j = i}^N \omega _{i,j}\sigma _{i,j}} \right)$$

where *σ*_*i,j*_ represents the number of instances of factor *i* and factor *j* bound to each other in that configuration (see below), *q*_*i*_ represents the change in free energy of RNA binding factor *i* binding to its respective site plus the natural log of the concentration of that factor, and *ω*_*i,j*_ is the change in free energy of factor *i* and factor *j* interacting, which is a measure of cooperativity between factor *i* and *j*. We assume that no factor binds to the spacer sequence and that all of the RNA-binding factors are present in the cell at roughly equal concentrations and bind 3′UTR with same affinity. We also assume that the rate limiting factor binds the 3′UTR with a particular affinity, set to two units for the PTRE-seq data. We model different factor-factor interactions by allowing cooperativity in particular configurations. In some cases, we allow cooperativity only when two factors are simultaneously bound to directly adjacent sites. In other cases, we allow cooperativity as long as the two factors are bound to the same 3′UTR at any two sites. The rate-limiting factor is allowed to interact with all factors bound to the 3′UTR. In our notation *q*_Factor_ and *ω*_Factor_ are equal, respectively, to ln(*q*_Factor_) and ln(*ω*_Factor_) described previously^51^. To calculate the probability that the rate-limiting factor is bound to the 3′UTR, and therefore the predicted level of the mRNA, we use the equation:

$${\rm mRNA}\;{\rm level} = \alpha \left( {\frac{{\mathop {\sum }\nolimits W \times \delta (\mathrm{RLF})}}{{\mathop {\sum }\nolimits W}}} \right)$$

where α is a scaling factor whose value is the least squares estimate, and *δ*(rate limiting factor) is a delta function that is zero when the rate limiting factor is not bound and one when it is bound. The summations are over all possible states of the 3′UTR. To fit the model to data we search for values of *α*, all *q*, and all *ω* that best fit the observed mRNA levels measured by PTRE-seq. The parameters are fit with custom Python scripts using SciPy to minimize the objective function using constrained minimization optimization algorithms L-BFGS-B and SLSQP in alternating fashion until the parameter values converge. The asymptotic normal distribution for the parameter estimate is used to calculate the 95% confidence intervals for the parameter values^46^. After fitting, any non-zero values of a particular *ω*_*i,j*_ are interpreted as cooperativity between the two factors.

### Data availability

The authors declare that the data supporting the findings described here are available within the article, the Supplementary Information or Supplementary Data 1–7. Extra data are available from the corresponding author upon request. Scripts used for analysis and model fitting are available at the Github repository under MIT license (https://github.com/hemangichaudhari/Cottrell_PTRE-seq_scripts). Sequencing data is available at the Sequence Read Archive under accession code SRP127467.

## Electronic supplementary material

Supplementary Information

Description of Additional Supplementary Files

Supplementary Data 1

Supplementary Data 2

Supplementary Data 3

Supplementary Data 4

Supplementary Data 5

Supplementary Data 6

Supplementary Data 7
