# Supplementary material for: PTRE-seq reveals mechanism and interactions of RNA binding proteins and miRNAs
Source: Nat Commun. 2018 Jan 19;9:301. doi: 10.1038/s41467-017-02745-0 (PMC5775260; doi:10.1038/s41467-017-02745-0)
Supplement: Supplementary file 1 — Supplementary Information [file 41467_2017_2745_MOESM1_ESM.pdf]

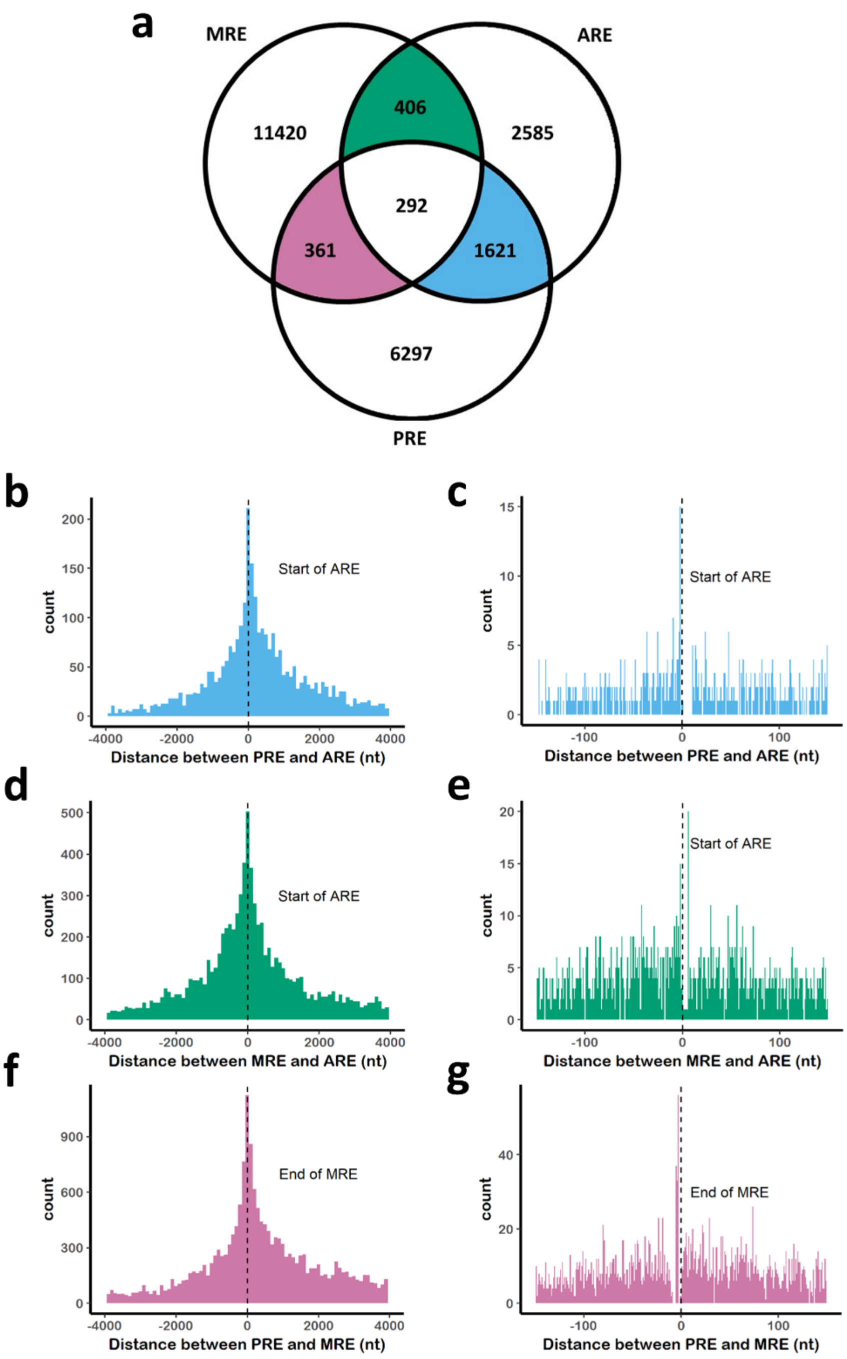

Supplementary Figure 1: The regulatory elements used in the library appear together in human transcripts. Venn-diagram showing the number of human mRNA transcripts that contain either a miRNA recognition element (MRE), PRE or ARE; and all combinations of those elements. Panels **b**-**g** show histograms of the distance between various mRNA regulatory elements. Distances are between the start of an ARE or PRE, or the end of an MRE. The locations of AREs were obtained from the ARE Database[1]. The locations of MREs were obtained from TargetScan[2]. We identified the locations of all PREs by a search for the consensus PRE: UGUAHAUA, where H is A, U or C, in all human 3'UTRs. Panels **b**, **d** and **f** show a broad view, +/- 4000 nt (bin-width of 100 nt) which is ~3*SD of the mean 3'UTR length in humans. Panels **c**, **e**, and **g** show a narrow view of +/- 150 nt (bin-width of 1 nt) which more closely resembles the size of the synthetic 3'UTRs used in the library. For MRE:ARE, 14.6% of all pairs are within 150 nt of each other. For PRE:ARE, 15.4% of all pairs are within 150 nt of each other. For MRE:PRE, 13.7% of all pairs are within 150 nt of each other.

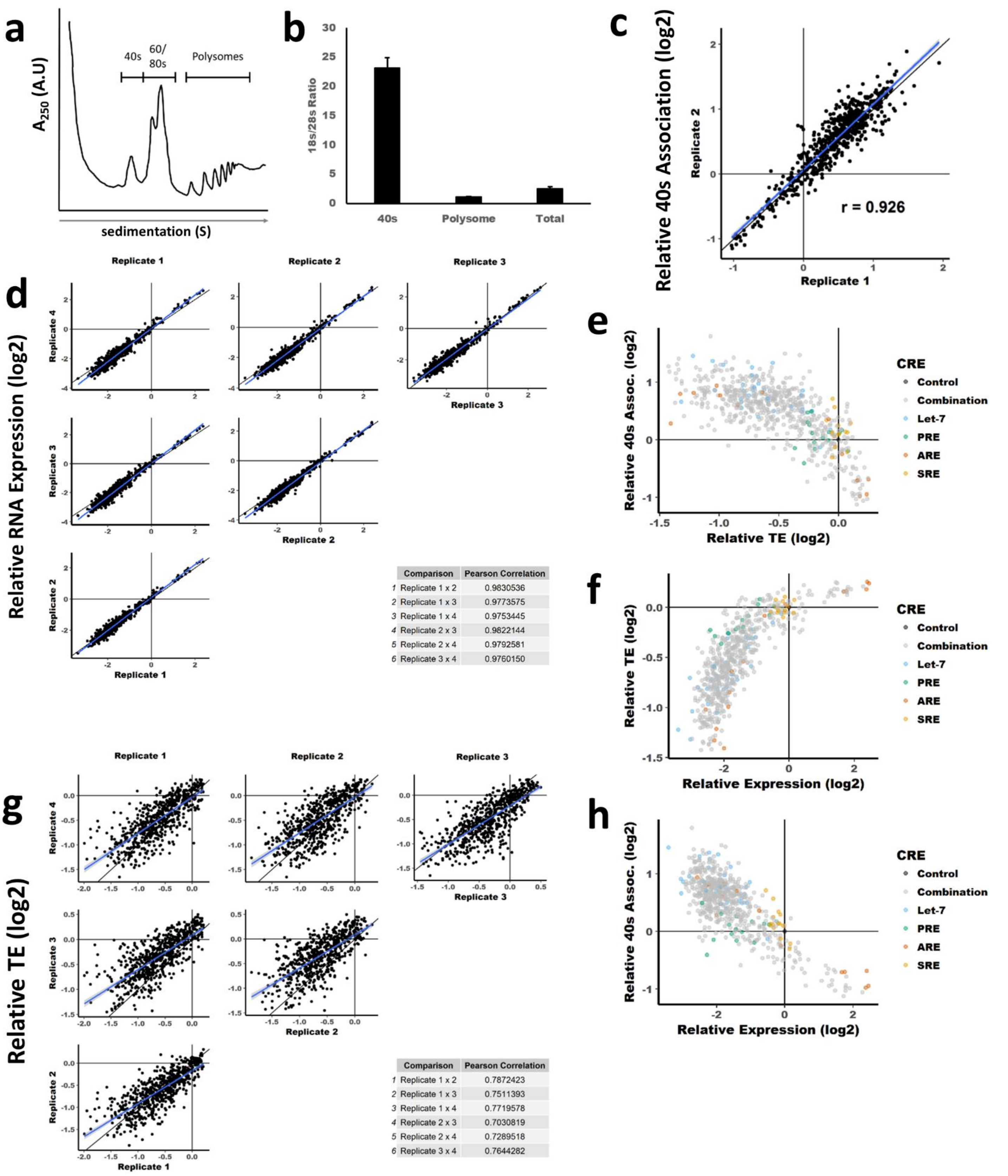

	Comparison	Pearson Correlation
1	Replicate 1 x 2	0.9830536
2	Replicate 1 x 3	0.9773575
3	Replicate 1 x 4	0.9753445
4	Replicate 2 x 3	0.9822144
5	Replicate 2 x 4	0.9792581
6	Replicate 3 x 4	0.9760150

	Comparison	Pearson Correlation
1	Replicate 1 x 2	0.7872423
2	Replicate 1 x 3	0.7511393
3	Replicate 1 x 4	0.7719578
4	Replicate 2 x 3	0.7030819
5	Replicate 2 x 4	0.7289518
6	Replicate 3 x 4	0.7644282

Supplementary Figure 2: The 40s fraction is enriched for the 18s rRNA of the small ribosomal subunit. **a** Representative polysome profiling trace. **b** qPCR was used to detect the abundance of the 18s and 28s rRNA in either the 40s fraction, the pooled polysome fractions or total RNA. Shown is the ratio of 18s/28s rRNA. Replicate to replicate comparisons of 40s association, **c**, relative RNA expression, **d**, and TE, **g**. The inset tables describe the Pearson correlation for each replicate to replicate comparison. Scatterplots of 40S association, RNA or TE, **e**, **f** and **h**.

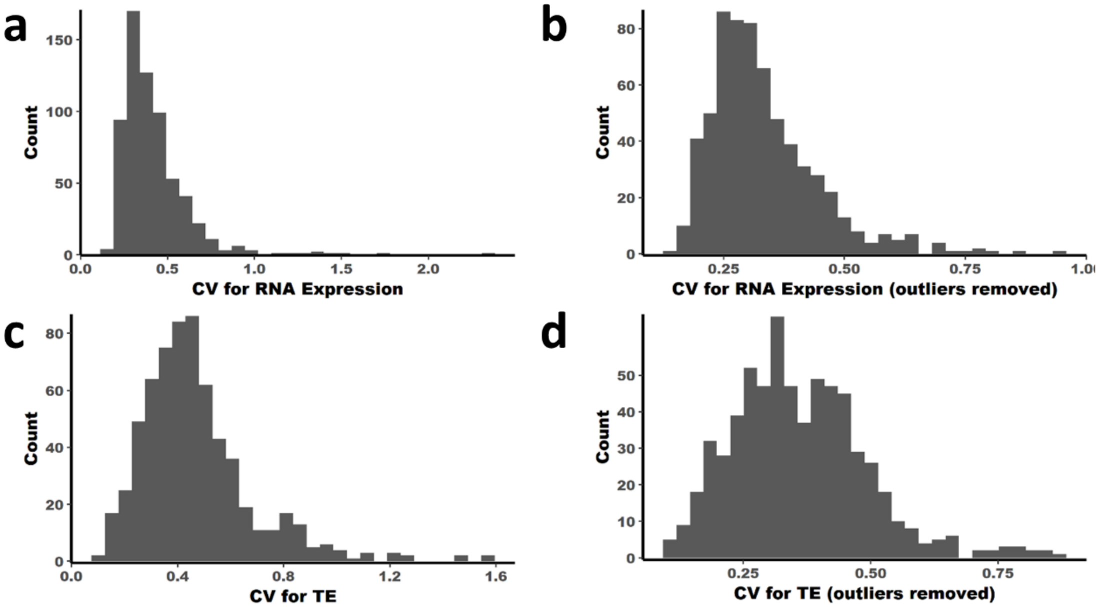

	RNA Expression	RNA Expression (outliers removed)	TE	TE (outliers removed)
**Median Fold Change**	-1.8435	-1.8407	-0.5321	-0.5204
**Median SD**	0.5382	0.4375	0.6423	0.4947
**Median SE**	0.0853	0.0707	0.1031	0.0813
**Median CV**	0.3696	0.3079	0.4330	0.3392
**Median Barcodes Detected/Reporter/ Replicate**	10	9.75	10	9.5

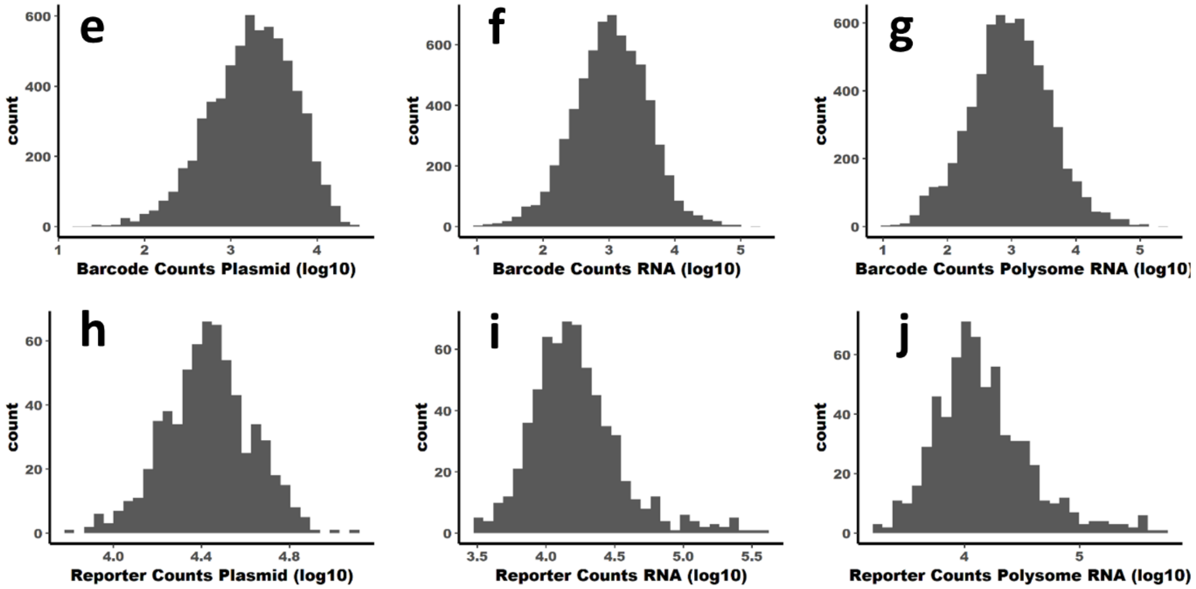

Supplementary Figure 3: Summary statistics for PTRE-seq analysis of RNA expression and TE. Histograms show the coefficient of variance (CV) across all barcode and replicates for each reporter within the library. Panel **a** shows the CV for RNA expression while panel **b** shows the CV for RNA expression with all outliers removed. Panel **c** shows the CV for TE while panel **d** shows the CV for TE with all outliers removed. Other summary statistics for RNA expression and TE measurements are shown in the table. Outliers were defined as any value more than 1.5 *IQR above Q1 or below Q3. Panels **e**-**j** show histograms of barcode counts (**e**, **f**, **g**) or reporter counts (sum of all barcode counts for each reporter; **h**, **i**, **j**) from one replicate of plasmid, RNA and polysome associated RNA sequencing.

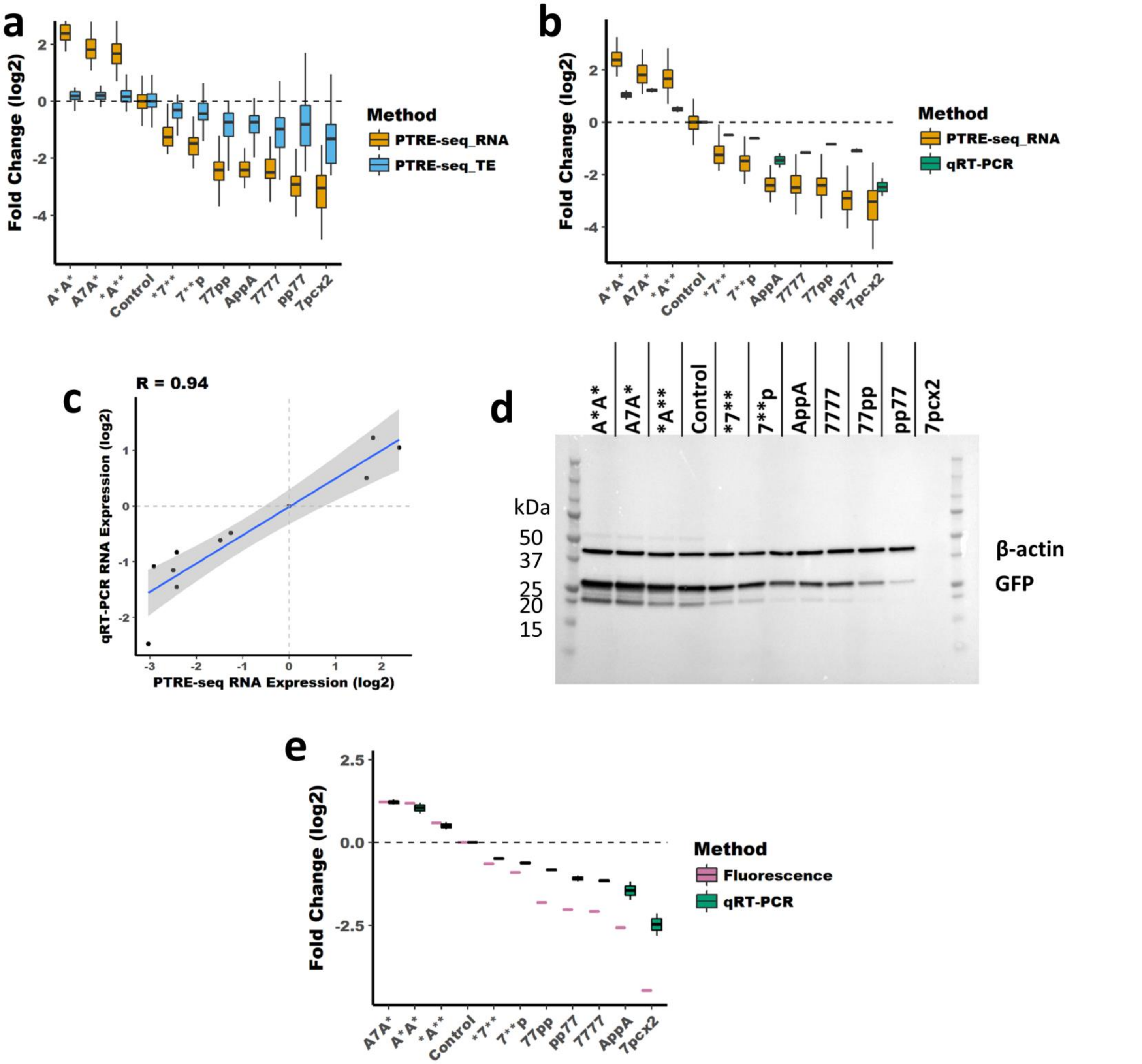

Supplementary Figure 4: Validation of PTRE-seq results by qPCR, fluorescence measurements and western blot analysis. We selected eleven reporters from our PTRE-seq library and transfected them individually into HeLa cells along with a plasmid for the expression of mCherry as a control, cells were harvested 40 hours later. Panel **a** shows the effect of the regulatory elements within these reporters as determined by PTRE-seq measurement of RNA expression and TE. Panel **b** shows a comparison between PTRE-seq measurement of RNA expression and qPCR of EGFP. For qPCR measurements of RNA expression, EGFP expression was normalized to mCherry. There is a strong correlation between PTRE-seq measurement of relative RNA expression and qRT-PCR of individual reporters, **c**. Panel **d** shows western blot analysis of EGFP and β-Actin. Panel **e** shows a comparison between qPCR measurements of RNA expression and fluorescent measurement of EGFP protein expression. For fluorescent measurements of EGFP expression the transfected cells were split into a 96-well plate the day after transfection. 40 hours after transfection the EGFP and mCherry fluorescence was measured using a plate reader. EGFP fluorescence was normalized to mCherry. For panels **a**, **b** and **d** relative RNA expression, TE or fluorescence was set relative to that of the control reporter, 4x Blank.

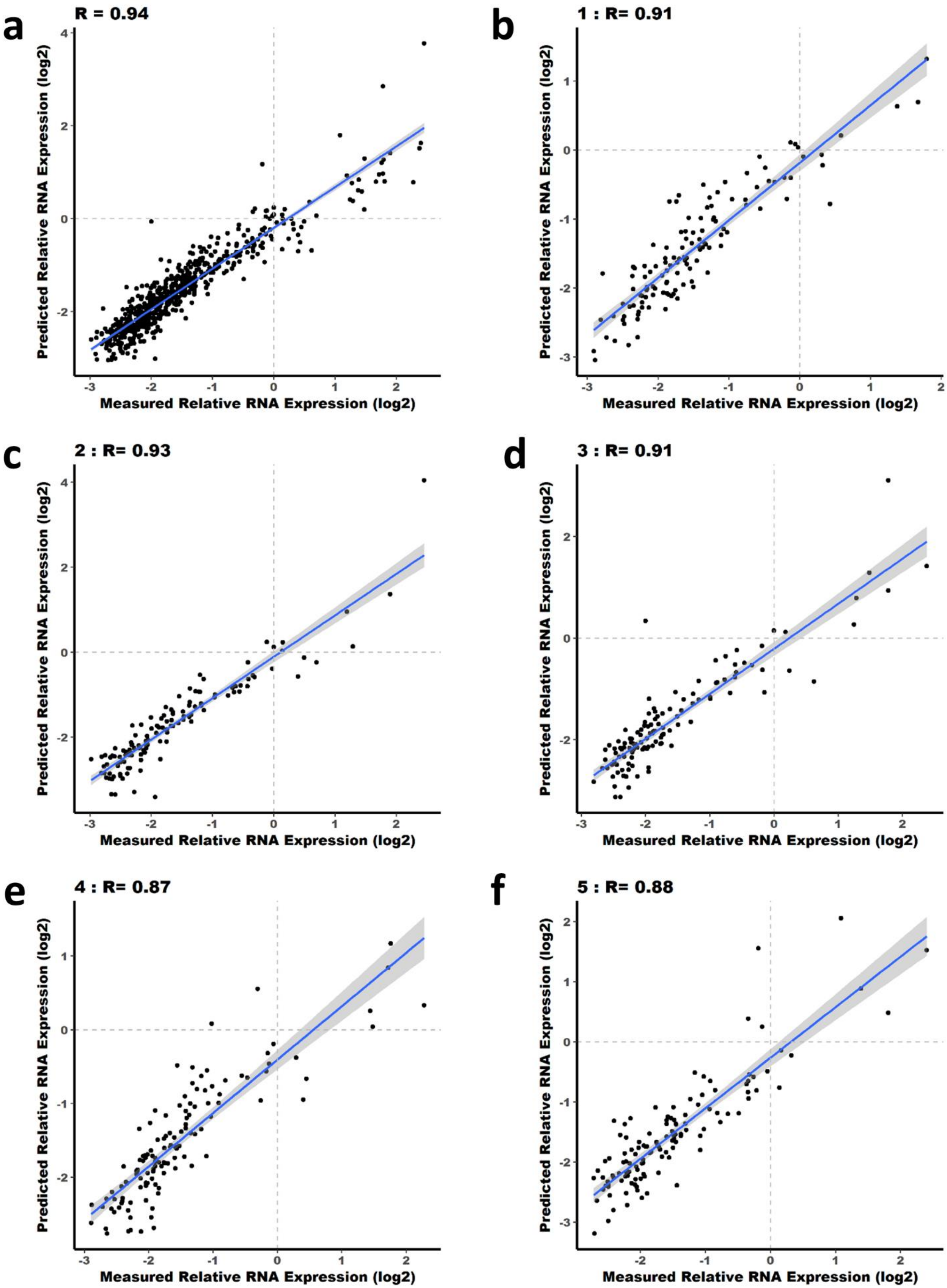

Supplementary Figure 5: **a** Fit of linear-regression model for RNA expression. Predicted relative RNA expression is plotted on the y-axis and measured relative RNA expression is plotted on the x-axis. The Pearson correlation coefficient shows a strong correlation between the predicted and measured RNA expression. **b-f** Five-fold cross validation of the model linear-regression model for RNA expression**.**

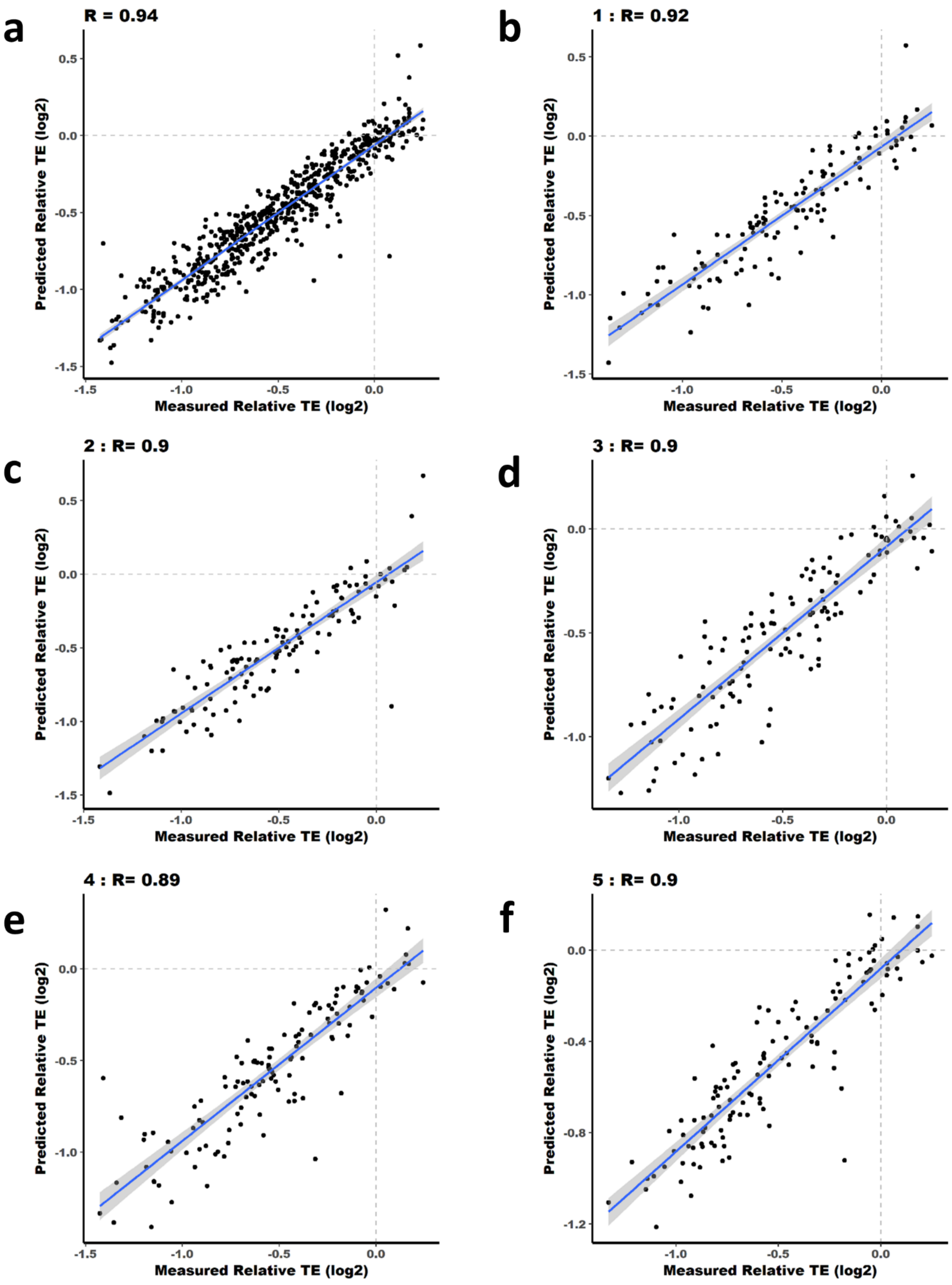

Supplementary Figure 6: a Fit of linear-regression model for TE. Predicted relative TE expression is plotted on the y-axis and measured relative TE expression is plotted on the x-axis. The Pearson correlation coefficient shows a strong correlation between the predicted and measured TE expression. **b-f** Five-fold cross validation of the model linear-regression model for TE**.**

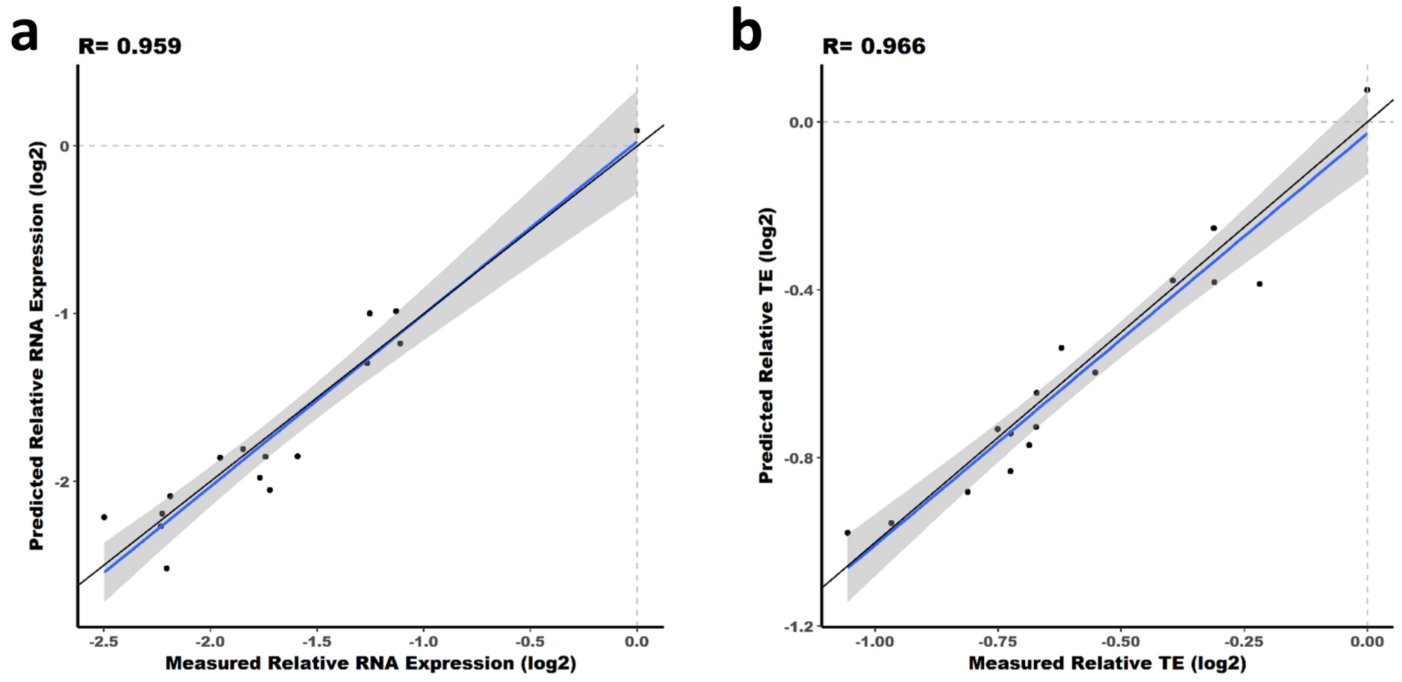

Regulatory Element	Correlation RNA Model	Correlation TE Model
Let-7	0.959466645	0.96599286
PRE	0.970774702	0.821543573
ARE	0.908129791	0.933569444
SRE	0.812835373	0.207737487
Let-7/PRE	0.955130642	0.937768779
Let-7/ARE	0.927219508	0.926926093
ARE/PRE	0.939196771	0.91296787

Supplementary Figure 7: Validation of the linear regression models for RNA and TE. The linear regression model described in Figure 1__ was used to predict the relative expression and TE of reporters containing only Let-7-target site, PRE, ARE, SRE and some combinations of those sites. The model predicted well the RNA expression, **a**, and TE, **b**, of reporters containing Let-7 binding sites. The correlations between predicted and measured RNA expression and TE for other 3'UTR elements and their combinations are shown in the table.

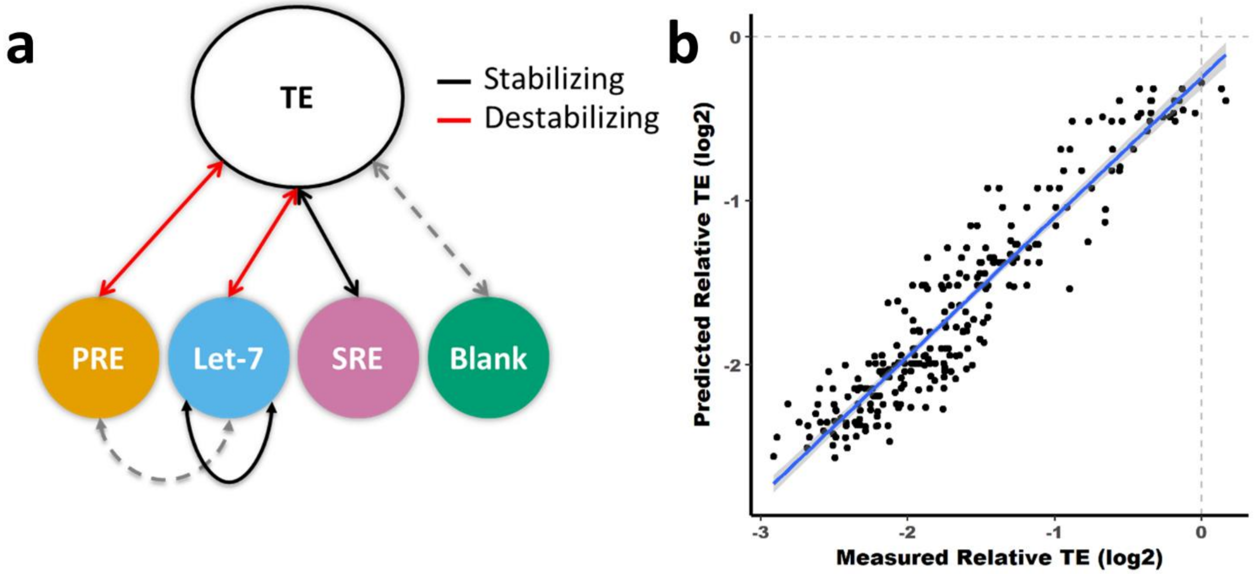

c

Parameter	Value (Proportional to free energy)	Significant
PRE : Ribosome	0.8513	Yes
Let-7 : Ribosome	4.103	Yes
SRE : Ribosome	-1.831	Yes
Blank : Ribosome	-2.859	No
PRE: Let-7, Let-7:PRE (neighboring)	-3.446	No
Let-7 : Let-7 (neighboring)	-4.126	Yes

Supplementary Figure 8: Thermodynamic model of contribution of PRE, *let-7* and SRE to TE. **a** A model with six parameters explained the observed data well (R^2 = 0.86). Red lines represent interactions with destabilizing effect on RNA. Black lines represent interactions with stabilizing effect on RNA. Solid lines represent statistically significant interactions and dashed lines represent non-significant interactions. Parameters values are in Supplementary Table 1. **b** Scatter plot shows the observed (x-axis) versus predicted (y-axis) normalized RNA counts from the model shown in panel A. **c** A table describing the parameters used in the model and their effects on TE.

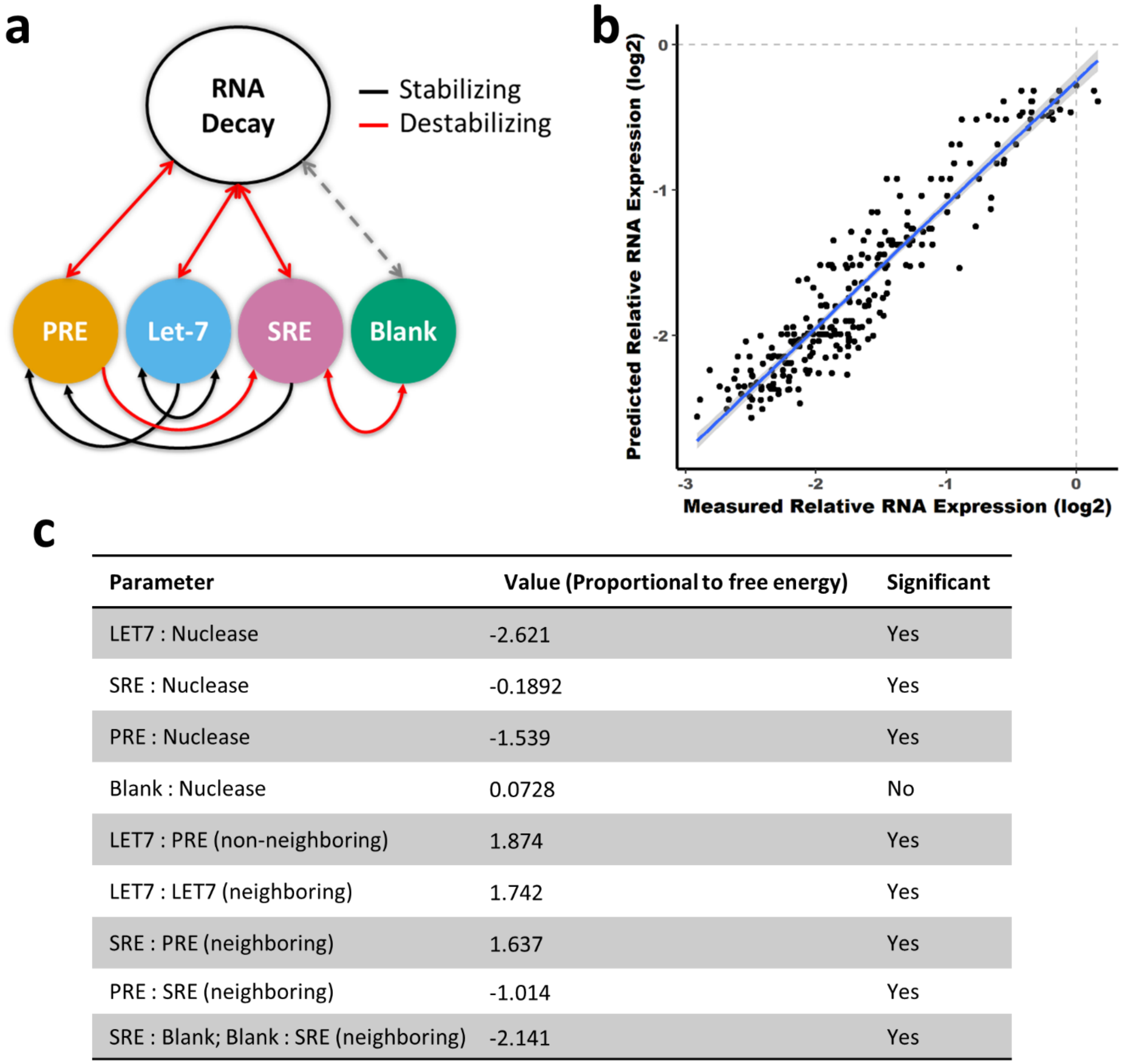

c

Parameter	Value (Proportional to free energy)	Significant
LET7 : Nuclease	-2.621	Yes
SRE : Nuclease	-0.1892	Yes
PRE : Nuclease	-1.539	Yes
Blank : Nuclease	0.0728	No
LET7 : PRE (non-neighboring)	1.874	Yes
LET7 : LET7 (neighboring)	1.742	Yes
SRE : PRE (neighboring)	1.637	Yes
PRE : SRE (neighboring)	-1.014	Yes
SRE : Blank; Blank : SRE (neighboring)	-2.141	Yes

Supplementary Figure 9: Thermodynamic model of contribution of PRE, *let-7* and SRE to RNA stability. **a** A model with nine parameters explained the observed data well (R^2 = 0.883). Red lines represent interactions with destabilizing effect on RNA. Black lines represent interactions with stabilizing effect on RNA. Solid lines represent statistically significant interactions and dashed lines represent non-significant interactions. Parameters values are in Supplementary Table 1. **b** Scatter plot shows the observed (x-axis) versus predicted (y-axis) normalized RNA counts from the model shown in panel A. **c** A table describing the parameters used in the model and their effects on RNA stability.

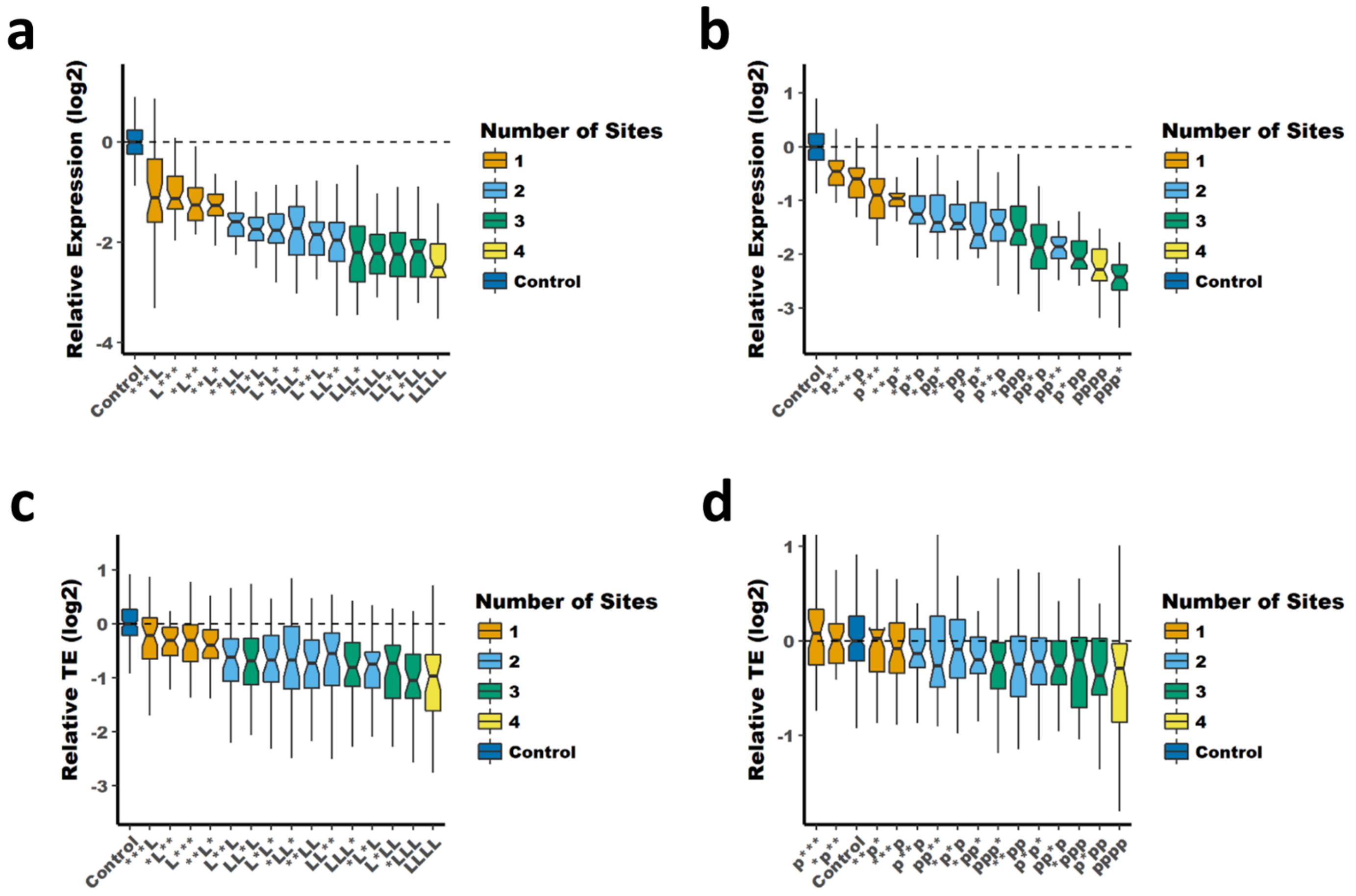

Supplementary Figure 10: Detailed results of *let-7* binding site containing reporters. Minimal position dependent effect of *let-7* binding site, **a**, or PRE, **b**, on RNA expression or TE, **c** and **d**. Fold repression of *let-7*, **e**, or PRE, **f**, containing reporters shown on a linear scale to highlight saturation of the effect on repression.

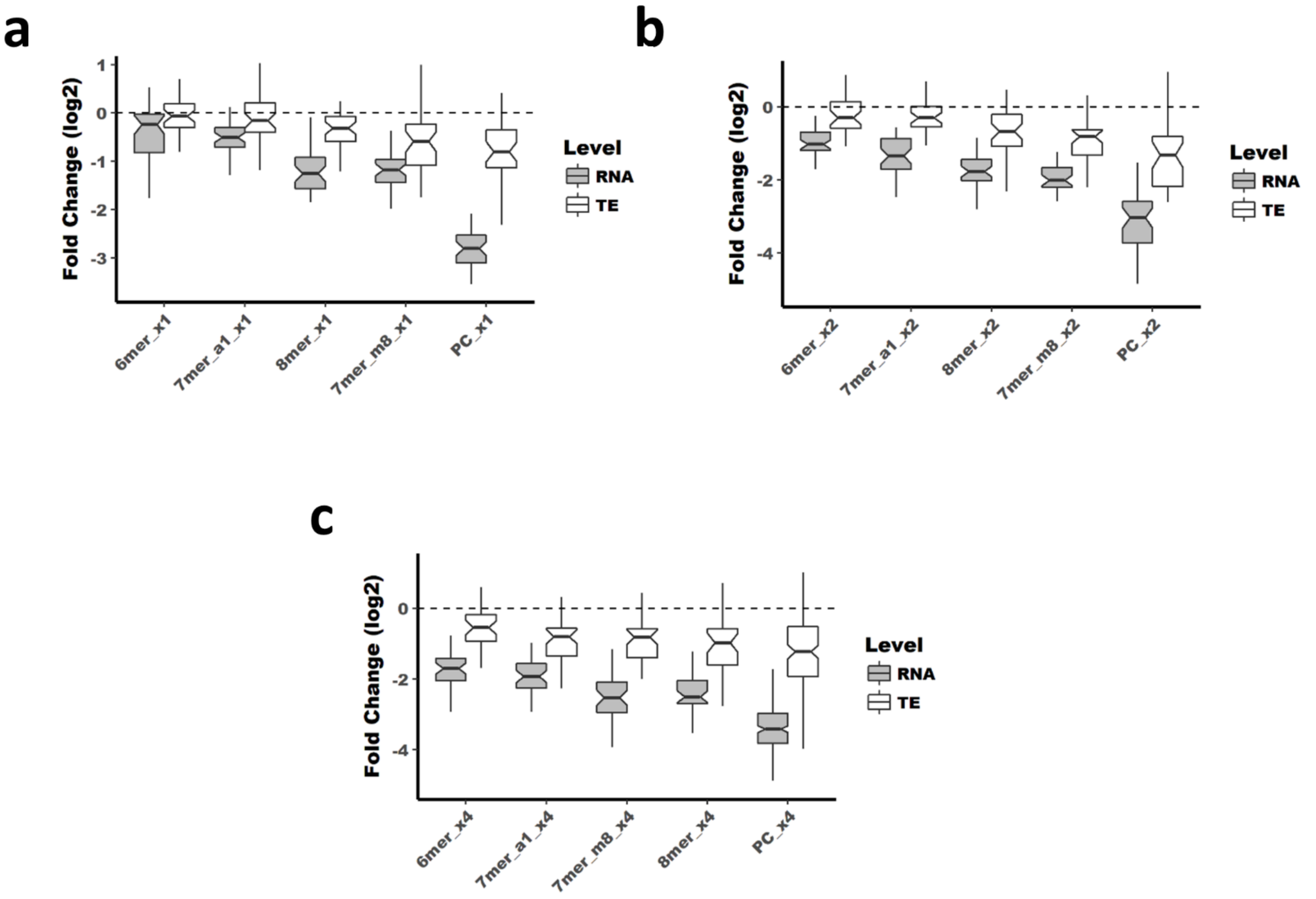

Supplementary Figure 11: The effect of seed pairing on repression of *let-7* targeted reporters containing one, **a**, two, **b**, or four, **c**, *let-7* binding sites.

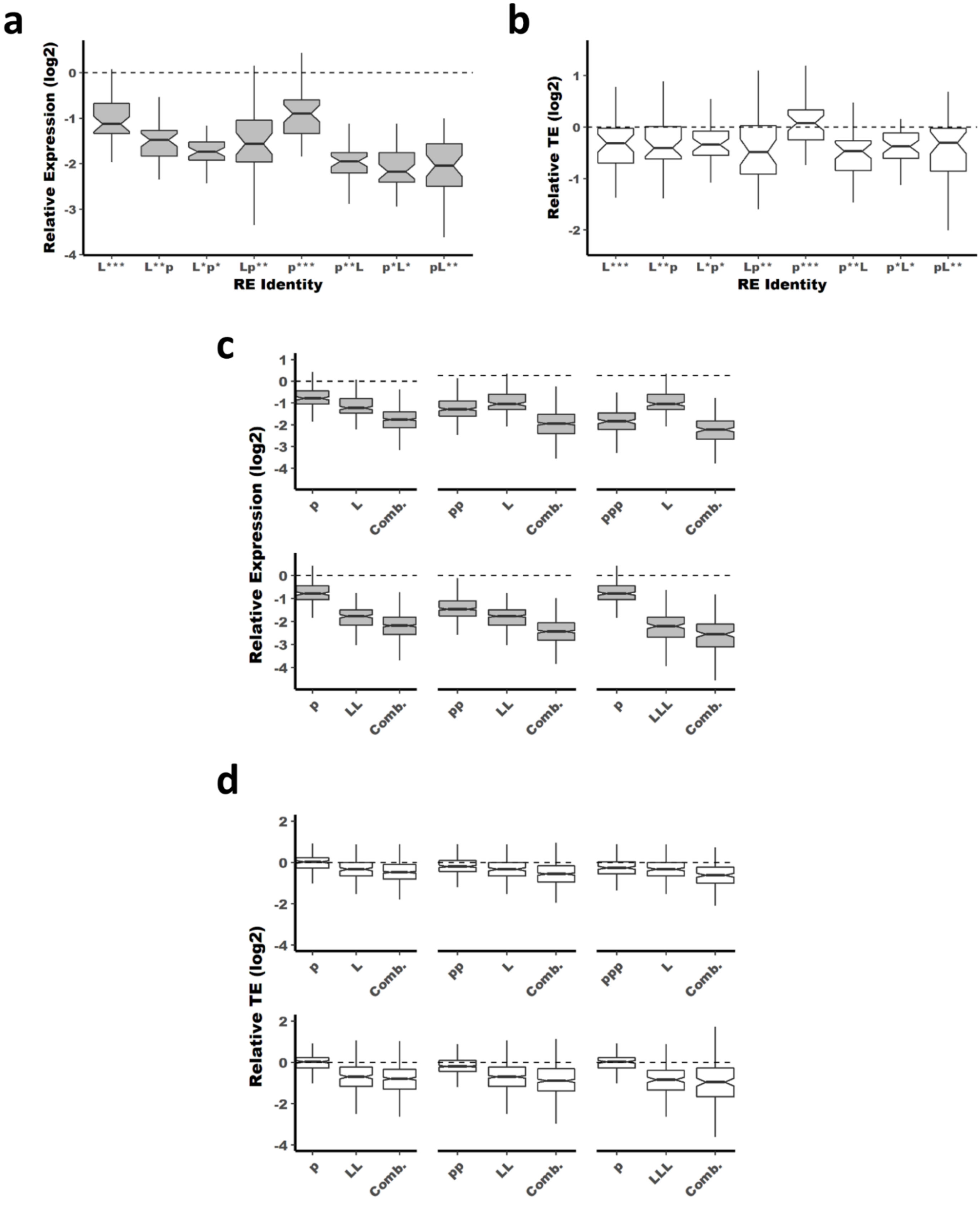

Supplementary Figure 12: The position of the *let-7* binding site or PRE has no effect on repression of RNA, **a**, or TE, **b**, when both elements are present in the 3'UTR. Panels **c** and **d** represent the same data plotted in Figure 4a and b as points.

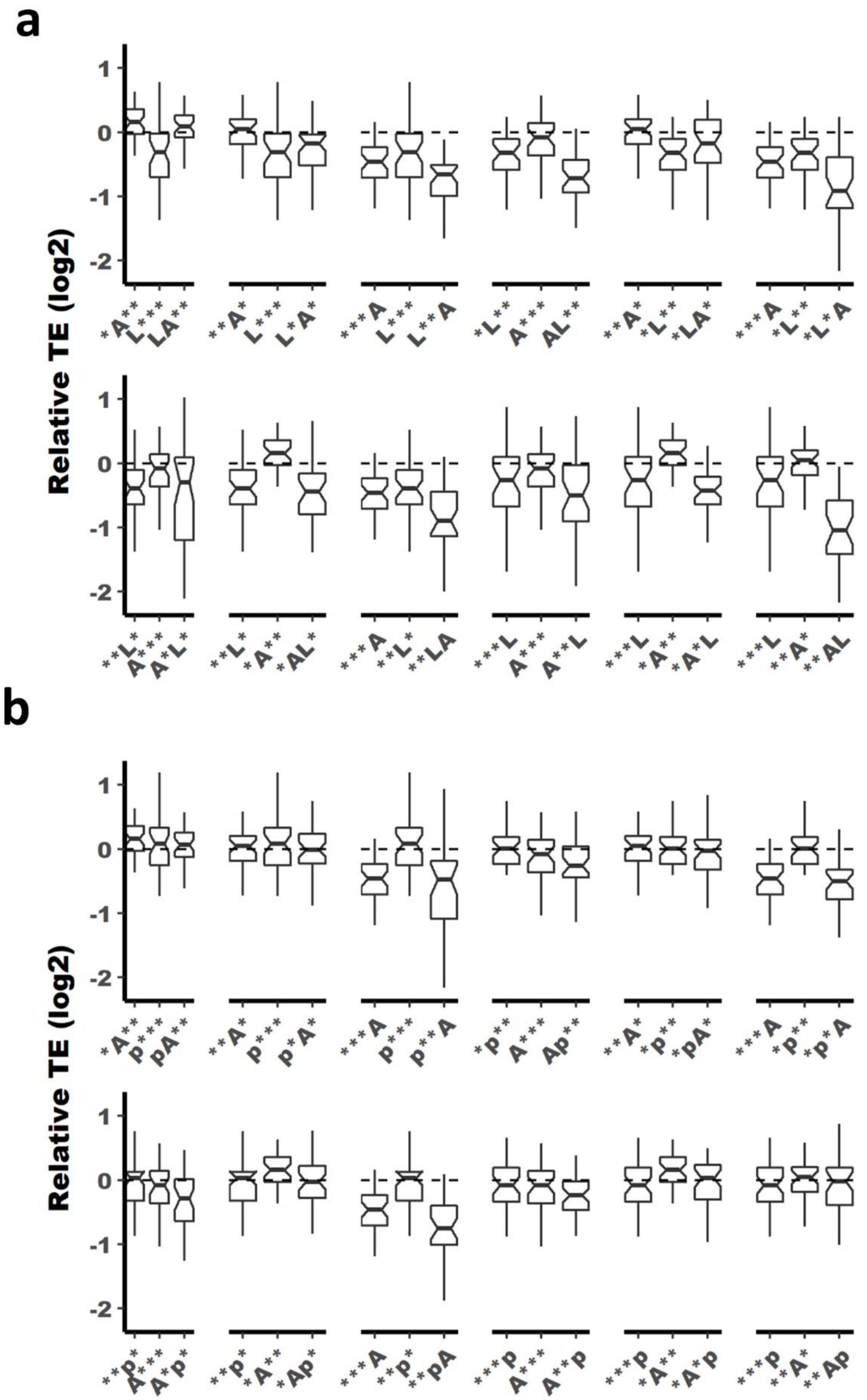

Supplementary Figure 14: a AREs modulate repression by miRNAs in a position dependent manner. **b** AREs modulate repression by PREs in a position dependent manner. * = Blank, A =ARE, L = *let-7* and p =PRE.

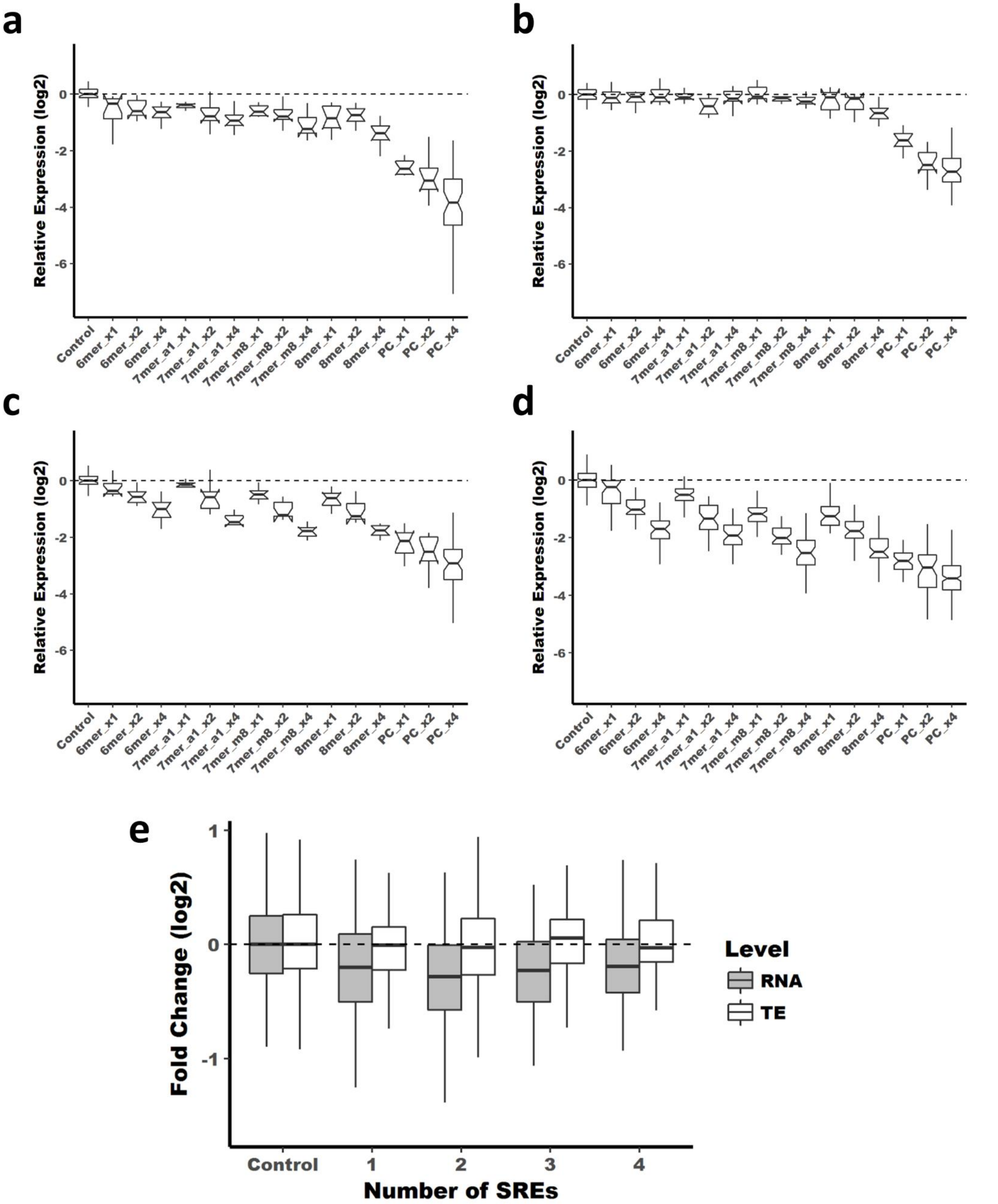

Supplementary Figure 15: The effect of seed sequence is on RNA expression is shown for each cell line tested, HDF, **a**, N2A, **b**, HEK, **c**, and HeLa, **d**. Fold change of RNA and TE for reporters containing SREs, **e**.

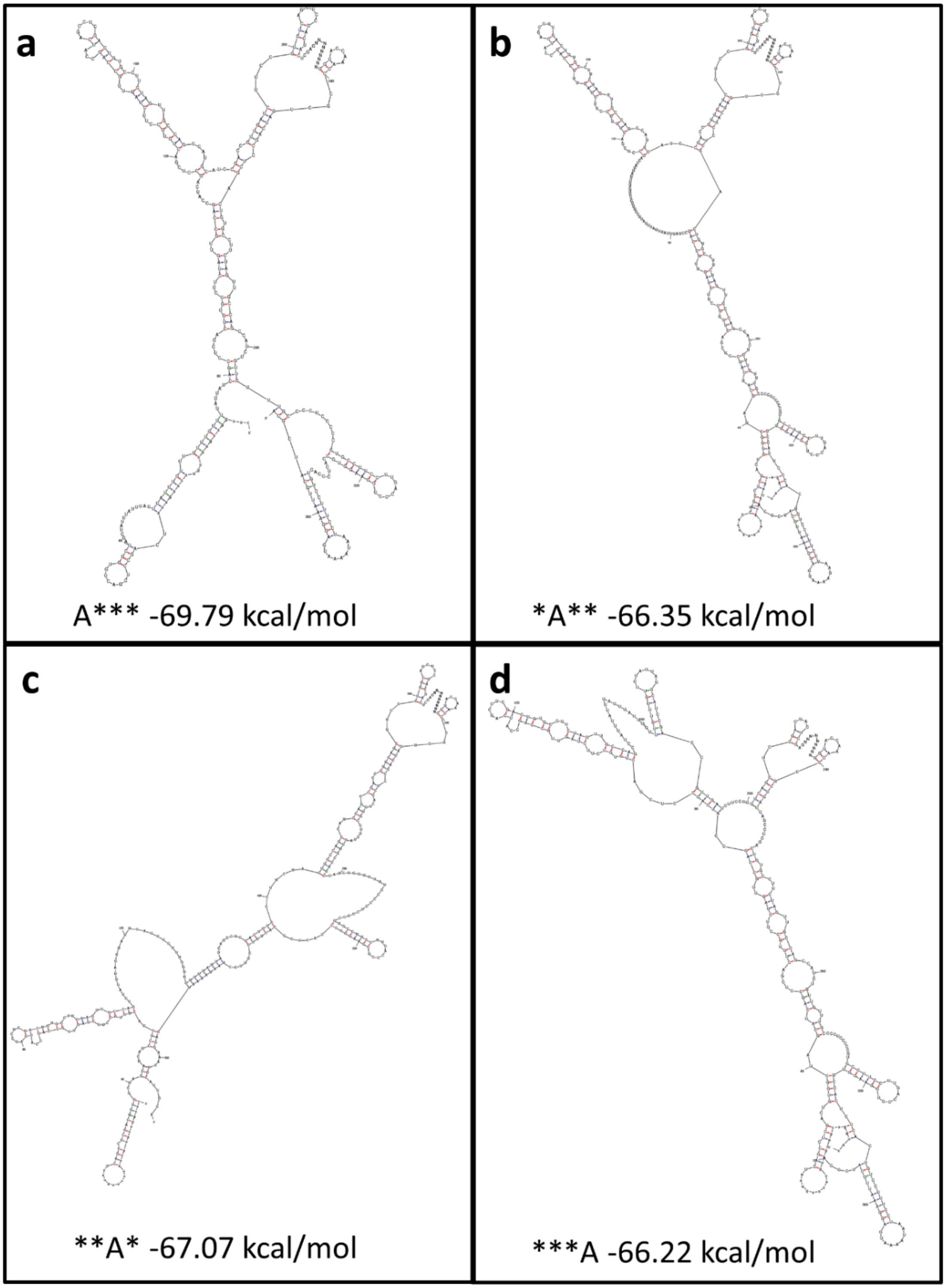

Supplementary Figure 16: Structure of the 3'UTR of reporters containing a single ARE in position one, **a**, two, **b**, three, **c**, or four, **d**. Structures were drawn using mfold[3].

Supplementary Table 1: Primers and Oligonucleotides

**Name**	**Sequence (5’-3’)**
EGFP-F	CACCATGGTGAGCAAGGGCGAGG
RE-R	GGTACCAGATATCTGCTAGCCACAGTCGAGGCTGATTACTTGTACAGCTCGTCCATG
Lib_F	GTAGCATCTGTCCGCTAGC
Lib_R	CGTAGTAGTAGTCGGGTACC
Spacer_F	TCCGACCGTTGATCTTCCGTGTCAGCTCCGACTAC
Spacer_R	TCGAGTAGTCGGAGCTGACACGGAAGATCAACGGTCGGATGCA
RE_Amp_F	GTTGATCTTCCGTGTCAGCTCC
RE_Amp_R	TGCAACTAGTAAGGACAGTGGGAGTGGCAC
Dme-Smg-Ovl-F	CAAGGGTGGCGGCGGTTCAATGAAGTACGCAACTGGAACTGAC
Dme-Smg-R	TTAGAATAGCGTAAAATGTTGATCAAATTTGG
mCh-F	CACCATGGGCGTGAGCAAGGGCGAGGAGG
mCh-Ovl-R	TGAACCGCCGCCACCCTTGTACAGCTCGTCCATGCC
18S rRNA F	GATGCCCTTAGATGTCCGGG
18S rRNA R	ATGGGGTTCAACGGGTTACC
28S rRNA F	AGTAACGGCGAGTGAACAGG
28S rRNA R	GCCTCGATCAGAAGGACTTG
P1-1_F	AATGATACGGCGACCACCGAGATCTACACTCTTTCCCTACACGACGCTCTTCCGATCTGCTCGAT
P1-1_R	/5Phos/<br>T*CGAATCGAGCAGATCGGAAGAGCGTCGTGTAGGGAAAGAGTGTAGATCTCGGTGGTCGCCGTATCATT
P1-2_F	AATGATACGGCGACCACCGAGATCTACACTCTTTCCCTACACGACGCTCTTCCGATCTTAGACTA
P1-2_R	/5Phos/<br>T*CGAATAGTCTAAGATCGGAAGAGCGTCGTGTAGGGAAAGAGTGTAGATCTCGGTGGTCGCCGTATCATT
P1-3_F	AATGATACGGCGACCACCGAGATCTACACTCTTTCCCTACACGACGCTCTTCCGATCTCGCTACCCT
P1-3_R	/5Phos/<br>T*CGAAGGGTAGCGAGATCGGAAGAGCGTCGTGTAGGGAAAGAGTGTAGATCTCGGTGGTCGCCGTATCATT
P1-4_F	AATGATACGGCGACCACCGAGATCTACACTCTTTCCCTACACGACGCTCTTCCGATCTATAGTGGACA
P1-4_R	/5Phos/<br>T*CGATGTCCACTATAGATCGGAAGAGCGTCGTGTAGGGAAAGAGTGTAGATCTCGGTGGTCGCCGTATCATT
P1-5_F	AATGATACGGCGACCACCGAGATCTACACTCTTTCCCTACACGACGCTCTTCCGATCTGTCAGTAGGTA
P1-5_R	/5Phos/<br>T*CGATACCTACTGACAGATCGGAAGAGCGTCGTGTAGGGAAAGAGTGTAGATCTCGGTGGTCGCCGTATCATT
P2-1_F	/5Phos/<br>C*TAGAGATCGGAAGAGCACACGTCTGAACTCCAGTCACCGCACTGGAATCTCGTATGCCGTCTTCTGCTTG
P2-1_R	CAAGCAGAAGACGGCATACGAGATTCCAGTGCGGTGACTGGAGTTCAGACGTGTGCTCTTCCGATCT
P2-2_F	/5Phos/<br>C*TAGAGATCGGAAGAGCACACGTCTGAACTCCAGTCACGTTGCAAGGATCTCGTATGCCGTCTTCTGCTTG
P2-2_R	CAAGCAGAAGACGGCATACGAGATCCTTGCAACGTGACTGGAGTTCAGACGTGTGCTCTTCCGATCT
Il_Enrich_F	AATGATACGGCGACCACCGAG
Il_Enrich_R	CAAGCAGAAGACGGCATACGA
EGFP_qF	AGGACGACGGCAACTACAAG
EGFP_qR	AAGTCGATGCCCTTCAGCTC
mCh_qF	CAAGGGCGAGGAGGATAACAT
mCh_qR	ACATGAACTGAGGGGACAGG

Control Oligo	GTAGCATCTGTCCGCTAGCATCAGCCTCGACTGTGCCTTCTAGTTGCCAGCCATCAGCCTCG ACTGTGCCTTCTAGTTGCCAGCCATCAGCCTCGACTGTGCCTTCTAGTTGCCAGCCATCAGC CTCGACTGTGCCTTCTAGTTGCCAGCCATGCATCGATATCACTCGAGGTGATCGCGGGTACC CGACTACTACTACG
*7** Oligo	GTAGCATCTGTCCGCTAGCATCAGCCTCGACTGTGCCTTCTAGTTGCCAGCCATCGAGACTA TACAAGGATCTACCTCAGTCGCAATCAGCCTCGACTGTGCCTTCTAGTTGCCAGCCATCAGC CTCGACTGTGCCTTCTAGTTGCCAGCCATGCATCGATATCACTCGAGAAGGCTCCTGGTACC CGACTACTACTACG
7777 Oligo	GTAGCATCTGTCCGCTAGCATCGAGACTATACAAGGATCTACCTCAGTCGCAATCGAGACTA TACAAGGATCTACCTCAGTCGCAATCGAGACTATACAAGGATCTACCTCAGTCGCAATCGAG ACTATACAAGGATCTACCTCAGTCGCAATGCATCGATATCACTCGAGTCCTGTATCGGTACC CGACTACTACTACG
7pcx2 Oligo	GTAGCATCTGTCCGCTAGCATCGAGACTATACAACCTACTACCTCAGTCGCAATCAGCCTCG ACTGTGCCTTCTAGTTGCCAGCCATCGAGACTATACAACCTACTACCTCAGTCGCAATCAGC CTCGACTGTGCCTTCTAGTTGCCAGCCATGCATCGATATCACTCGAGCTAATCCACGGTACC CGACTACTACTACG

Supplementary Table 2: Binding Sites for RBP and miRNA

**Regulatory Element**	**Sequence**	**Source**
Let-7 binding site	aUCGAGACUAUACAAGGAUCUACCUCAGUCGca	Synthetic [4]
AU-rich Element (ARE)	UAUUUAUUUAUUUAUUUGUUUGUUUGUUUUAUU	IL-1β 3'UTR [5]
Pumilio Recognition Element (PRE)	gucagcuccgacuUGUAAAUAucagccucgacu	Consensus binding site [6]
Smaug Recognition Element (SRE)	cacaaGCAGAGGCUCUGGCAGCUUUUGCcaaca	Nanos 3'UTR (*Dme*) [7]
Blank	aucagccucgacugugccuucuaguugccagcc	BGH 3'UTR, pcDNA5/FRT/TO (Invitrogen)

Supplementary References

1. Bakheet, T., Hitti, E. & Khabar, K.S.A. ARED-Plus: an updated and expanded database of AU-rich element-containing mRNAs and pre-mRNAs. *Nucleic Acids Res* (2017).
2. Agarwal, V., Bell, G.W., Nam, J.W. & Bartel, D.P. Predicting effective microRNA target sites in mammalian mRNAs. *Elife* **4** (2015).
3. Zuker, M. Mfold web server for nucleic acid folding and hybridization prediction. *Nucleic Acids Res* **31**, 3406-3415 (2003).
4. Iwasaki, S., Kawamata, T. & Tomari, Y. Drosophila argonaute1 and argonaute2 employ distinct mechanisms for translational repression. *Mol Cell* **34**, 58-67 (2009).
5. Meisner, N.C. et al. mRNA openers and closers: modulating AU-rich element-controlled mRNA stability by a molecular switch in mRNA secondary structure. *Chembiochem* **5**, 1432-1447 (2004).
6. Campbell, Z.T., Valley, C.T. & Wickens, M. A protein-RNA specificity code enables targeted activation of an endogenous human transcript. *Nat Struct Mol Biol* **21**, 732-738 (2014).
7. Smibert, C.A., Lie, Y.S., Shillinglaw, W., Henzel, W.J. & Macdonald, P.M. Smaug, a novel and conserved protein, contributes to repression of nanos mRNA translation in vitro. *RNA* **5**, 1535-1547 (1999).
