# Supplementary material for: PTRE-seq reveals mechanism and interactions of RNA binding proteins and miRNAs
Source: Nat Commun. 2018 Jan 19;9:301. doi: 10.1038/s41467-017-02745-0 (PMC5775260; doi:10.1038/s41467-017-02745-0)
Supplement: Supplementary file 2 — Description of Additional Supplementary Files [file 41467_2017_2745_MOESM2_ESM.pdf]

Description of Additional Supplementary Files

File Name: Supplementary Data 1
Description: File contains the synthetic 3'UTR sequences used in the library.

File Name: Supplementary Data 2
Description: The number sequencing reads corresponding to each barcode/synthetic 3'UTR in plasmid sample.

File Name: Supplementary Data 3
Description: The number sequencing reads corresponding to each barcode/synthetic 3'UTR in four HeLa cells replicates.

File Name: Supplementary Data 4
Description: The number sequencing reads corresponding to each barcode/synthetic 3'UTR in polysome associated fractions from four HeLa cell replicates.

File Name: Supplementary Data 5
Description: The number sequencing reads corresponding to each barcode/synthetic 3'UTR in 40S subunit associated fractions from four HeLa cell replicates.

File Name: Supplementary Data 6
Description: The number sequencing reads corresponding to each barcode/synthetic 3'UTR in RNA samples from different cell lines (N2A, Hek293 and HDF)

File Name: Supplementary Data 7
Description: The number sequencing reads corresponding to each barcode/synthetic 3'UTR in RNA samples from mCherry-Smaug transfected HeLa cells
